# Directed Evolution Reveals Unexpected Epistatic Interactions That Alter Metabolic Regulation and Enable Anaerobic Xylose Use by *Saccharomyces cerevisiae*

**DOI:** 10.1371/journal.pgen.1006372

**Published:** 2016-10-14

**Authors:** Trey K. Sato, Mary Tremaine, Lucas S. Parreiras, Alexander S. Hebert, Kevin S. Myers, Alan J. Higbee, Maria Sardi, Sean J. McIlwain, Irene M. Ong, Rebecca J. Breuer, Ragothaman Avanasi Narasimhan, Mick A. McGee, Quinn Dickinson, Alex La Reau, Dan Xie, Mingyuan Tian, Jennifer L. Reed, Yaoping Zhang, Joshua J. Coon, Chris Todd Hittinger, Audrey P. Gasch, Robert Landick

**Affiliations:** 1 DOE Great Lakes Bioenergy Research Center, University of Wisconsin-Madison, Madison, Wisconsin, United States of America; 2 Genome Center of Wisconsin, University of Wisconsin-Madison, Madison, Wisconsin, United States of America; 3 Laboratory of Genetics, University of Wisconsin-Madison, Madison, Wisconsin, United States of America; 4 Department of Chemistry, University of Wisconsin-Madison, Madison, Wisconsin, United States of America; 5 Microbiology Doctoral Training Program, University of Wisconsin-Madison, Madison, Wisconsin, United States of America; 6 Department of Chemical and Biological Engineering, University of Wisconsin-Madison, Madison, Wisconsin, United States of America; 7 Department of Biomolecular Chemistry, University of Wisconsin-Madison, Madison, Wisconsin, United States of America; 8 Wisconsin Energy Institute, J.F. Crow Institute for the Study of Evolution, University of Wisconsin-Madison, Madison, Wisconsin, United States of America; 9 Department of Biochemistry, University of Wisconsin-Madison, Madison, Wisconsin, United States of America; University of Toronto, CANADA

## Abstract

The inability of native *Saccharomyces cerevisiae* to convert xylose from plant biomass into biofuels remains a major challenge for the production of renewable bioenergy. Despite extensive knowledge of the regulatory networks controlling carbon metabolism in yeast, little is known about how to reprogram *S*. *cerevisiae* to ferment xylose at rates comparable to glucose. Here we combined genome sequencing, proteomic profiling, and metabolomic analyses to identify and characterize the responsible mutations in a series of evolved strains capable of metabolizing xylose aerobically or anaerobically. We report that rapid xylose conversion by engineered and evolved *S*. *cerevisiae* strains depends upon epistatic interactions among genes encoding a xylose reductase (*GRE3*), a component of MAP Kinase (MAPK) signaling (*HOG1*), a regulator of Protein Kinase A (PKA) signaling (*IRA2*), and a scaffolding protein for mitochondrial iron-sulfur (Fe-S) cluster biogenesis (*ISU1*). Interestingly, the mutation in *IRA2* only impacted anaerobic xylose consumption and required the loss of *ISU1* function, indicating a previously unknown connection between PKA signaling, Fe-S cluster biogenesis, and anaerobiosis. Proteomic and metabolomic comparisons revealed that the xylose-metabolizing mutant strains exhibit altered metabolic pathways relative to the parental strain when grown in xylose. Further analyses revealed that interacting mutations in *HOG1* and *ISU1* unexpectedly elevated mitochondrial respiratory proteins and enabled rapid aerobic respiration of xylose and other non-fermentable carbon substrates. Our findings suggest a surprising connection between Fe-S cluster biogenesis and signaling that facilitates aerobic respiration and anaerobic fermentation of xylose, underscoring how much remains unknown about the eukaryotic signaling systems that regulate carbon metabolism.

## Author Summary

The yeast *Saccharomyces cerevisiae* is being genetically engineered to produce renewable biofuels from sustainable plant material. Efficient biofuel production from plant material requires conversion of the complex suite of sugars found in plant material, including the five-carbon sugar xylose. Because it does not efficiently metabolize xylose, *S*. *cerevisiae* has been engineered with a minimal set of genes that should overcome this problem; however, additional genetic changes are required for optimal fermentative conversion of xylose into biofuel. Despite extensive knowledge of the regulatory networks controlling glucose metabolism, less is known about the regulation of xylose metabolism and how to rewire these networks for effective biofuel production. Here we report genetic mutations that enabled the conversion of xylose into bioethanol by a previously ineffective yeast strain. By comparing altered protein and metabolite abundance within yeast cells containing these mutations, we determined that the mutations synergistically alter metabolic pathways to improve the rate of xylose conversion. One change in a gene with well-characterized aerobic mitochondrial functions was found to play an unexpected role in anaerobic conversion of xylose into ethanol. The results of this work will allow others to rapidly generate yeast strains for the conversion of xylose into biofuels and other products.

## Introduction

Biofuels, such as ethanol, produced by microbial fermentation of plant-derived feedstocks offer renewable, carbon-neutral forms of energy. Lignocellulosic hydrolysates are generated by chemical pretreatment and hydrolysis of plant cell walls, which are composed of lignin, cellulose, and hemicellulose, and contain glucose, xylose, other carbohydrates, and diverse small molecules. *Saccharomyces cerevisiae*, the predominant microbe used by the starch ethanol industry, excels at fermenting glucose, but lacks both sufficient metabolic activities and appropriate regulatory responses to ferment xylose rapidly and efficiently [1]. To become economically feasible, microbes must be able to ferment the complete suite of sugars including xylose, which can be up to half of the total fermentable sugar in some lignocellulosic hydrolysates.

In order to achieve a minimal level of xylose catabolism, yeasts have been engineered to express the xylose isomerase (XI)-xylulokinase (XK) pathway or the xylose reductase-xylitol dehydrogenase-xylulokinase pathway to produce xylulose-5-phosphate (X5P), which can then be further converted via the pentose phosphate and glycolytic pathways into ethanol (reviewed in [2–5]). Improved xylose-fermenting *S*. *cerevisiae* strains were the result of intensive rational engineering to over-express additional metabolic enzymes [6, 7]. Directed evolution has further improved strains to achieve greater fermentative capacity for xylose (reviewed in [1]). However the underlying genetic mechanisms of xylose fermentation remain largely unexplored. To date, three separate studies reported the identities of evolved mutations directly linked to improved xylose metabolism. These include evolved mutations in the alkaline phosphatase *PHO13*, which was implicated in xylose catabolism through transposon library screening [8], the hexose transporter *HXT7*, which caused an increased xylose uptake rate [9], and *GRE3* [10], which encodes an aldose reductase that converts xylose into xylitol [11, 12], an inhibitor of xylose isomerase [13]. Even with these genetic modifications, *S*. *cerevisiae* strains do not achieve industrially acceptable xylose fermentation rates, indicating that additional metabolic and regulatory bottlenecks limit xylose conversion.

In contrast to our limited understanding of xylose metabolism, the regulatory systems that control glucose assimilation in *S*. *cerevisiae* are among the best-understood networks in eukaryotic cells. Yeast sense and respond to a range of glucose concentrations through multiple signaling pathways that regulate specific transcriptional and metabolic responses. This tight regulatory response to glucose enables *S*. *cerevisiae* to be one of few organisms that ferment glucose into ethanol aerobically through high glycolytic flux (reviewed in [14]). Three signaling pathways mediated by cyclic AMP (cAMP)-Protein Kinase A (PKA), Snf3/Rgt2, and Snf1 are primarily responsible for coordinating this response (recently reviewed in [15–21]). Glucose sensing by the G-protein coupled receptor Gpr1p and Ras GTPase activate production of cAMP by adenylate cyclase, which subsequently stimulates PKA activity [22]. Activated PKA has both positive and negative regulatory functions; phosphorylation of cytosolic targets causes activation of glycolysis [23, 24] and other metabolic pathways, whereas phosphorylation of transcription factors causes repression of genes involved in stress response [25] and in the metabolism of non-fermentable carbon substrates [26], such as oxidation of ethanol. Slightly less well understood is the pathway mediated by the paralogous transmembrane sensors Snf3p and Rgt2. Snf3p senses low concentrations of glucose, while Rgt2p acts as a sensor for high glucose concentrations [27, 28]. These sensors fine-tune the expression of a large family of hexose transporters (*HXT*), which display a range of affinities for binding and transporting glucose according to its extracellular availability. Lastly, the AMP-activated kinase (AMPK) Snf1p is the third rheostat controlling the response to glucose. In the absence of glucose, Snf1p is active and promotes the expression of genes and activation of proteins involved in respiratory metabolism, gluconeogenesis, and the glyoxylate cycle, while repressing anabolic processes [15, 18]. In the presence of glucose, Snf1p is inactive such that metabolism of ethanol, glycerol, acetate and other non-preferred carbon sources are repressed. Only upon depletion of glucose or other fermentable sugars (*e*.*g*., fructose) does *S*. *cerevisiae* undergo diauxic shift to respire ethanol or other non-fermentable carbon substrates. Through this complex interplay of signaling networks, *S*. *cerevisiae* is able to achieve rapid conversion of glucose into ethanol.

Despite this extensive understanding of glucose metabolism and numerous research efforts, it remains unclear how to reprogram regulatory networks in *S*. *cerevisiae* to convert xylose into ethanol or other biofuels rapidly and efficiently. Here, we report novel epistatic genetic interactions between mutations in genes involved in MAPK (*HOG1*) and cAMP-PKA (*IRA2*) signaling pathways, assembly and transfer of Fe-S clusters (*ISU1*) and *GRE3* that collectively enable xylose metabolism under various oxygen conditions. Using proteomic and metabolomic analyses, we discovered that loss of *ISU1* function is crucial for aerobic respiration and anaerobic fermentation of xylose, and that epistatic interactions with *IRA2* mutations are essential for anaerobic fermentation. Based on the individual effects of these mutations on protein and metabolite levels and on use of xylose and other carbon sources, we propose a mechanistic model to explain their effects. Our findings have major implications for the understanding of the pathways controlling nutrient signaling and contribute towards improving metabolic engineering for the production of lignocellulosic biofuels.

## Results

### Mutations in *ISU1* and *HOG1* underlie aerobic xylose fermentation

**Fig 1:**
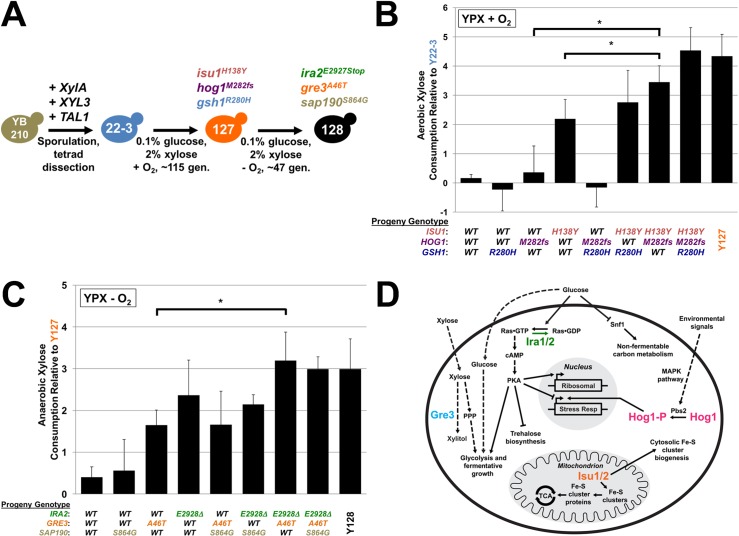
Mutations in *ISU1*, *HOG1*, *GRE3* and *IRA2* co-segregate with the evolved xylose metabolism phenotypes. The schematic diagram in (**A**) summarizes the genetic engineering and evolution of the engineered and evolved strains used in this study. Evolved strains were backcrossed to their corresponding ancestral strain and resulting progeny were genotyped and phenotyped for their abilities to consume xylose from lab media relative to the ancestor. Bar graphs represent average Log_2_ fold-differences of xylose consumed by individual spores from Y22-3 x Y127 (**B**) or Y127 x Y128 (**C**) backcrosses relative to their parental strains. Average differences and standard deviations were determined from 2–5 independently generated spores in biological triplicate growth experiments. Asterisks (*) denote statistical significance between indicated strains by Student’s t-test, *P* < 0.05. *WT*; wild-type. The schematic diagram (**D**) depicts some known cellular functions of yeast *HOG1*, *IRA* and *ISU*. Arrowheads indicate interactions of positive or negative regulation.

Previously, we described the generation and characterization of a series of engineered and sequentially evolved *S*. *cerevisiae* strains with a range of abilities to consume and metabolize xylose: (i) GLBRCY22-3 (Y22-3), which was generated from a genetically engineered monosporic derivative of the stress-tolerant NRRL YB-210 *S*. *cerevisiae* strain [29]; (ii) GLBRCY127 (Y127), which is a single clone isolated from the aerobic directed evolution of Y22-3 on xylose; and (iii) GLBRCY128 (Y128), a single clone isolated from the anaerobic directed evolution of Y127 on xylose (**Fig 1A** and [10]). Here, we set out to define the mutations responsible for improved xylose metabolism at each stage in the evolutionary trajectory, from the parental strain Y22-3, to aerobic xylose-consuming Y127, and then to anaerobic xylose-fermenting Y128. To define these mutations, we first mapped Illumina sequence reads from Y127 and Y128 genomes to the sequenced and assembled Y22-3 parental genome [30], and then identified both single nucleotide polymorphisms (SNPs) and DNA insertion/deletion (indel) mutations that arose during the directed evolution (see Materials and Methods). In the Y127 strain, which can rapidly metabolize xylose aerobically, we found non-synonymous SNPs in *ISU1* and *GSH1*, which encode a mitochondrial iron-sulfur (Fe-S) cluster chaperone and **γ**-glutamylcysteine synthetase, respectively, a single base-pair, frame-shifting deletion in the Mitogen Activated Protein Kinase (MAPK) *HOG1*, and a single base-pair insertion in a Ty element within the left arm subtelomere of Chromosome XIV (**Table 1**). The *hog1*^*M282fs*^ mutation is predicted to generate a scrambled sequence of 31 amino acids before terminating well short of the 435 amino acids for wild-type Hog1p. Under osmotic and other environmental stresses, Hog1p is phosphorylated by Pbs2p and then translocates into the nucleus to regulate transcription of stress response genes (reviewed in [31]). The mutation *isu1*^*H138Y*^ substitution resides adjacent to a functionally important tripeptide domain [32]. *ISU1* and its paralog *ISU2* encode mitochondrial-localized proteins involved in assembling Fe-S clusters, which are co-factors for proteins involved in electron transfer, enzymatic reactions, and oxygen sensing [33, 34].

**Table 1:** Genetic differences between parental and evolved strains.

Evolved Strain	Parental Strain	Gene	Functional Gene Annotation^1^	Nucleotide Difference^2^	Amino Acid Difference^3^
Y127	Y22-3	*ISU1*	Fe-S cluster assembly	C412T	H138Y
Y127	Y22-3	*HOG1*	MAP kinase signaling	A844del	M282frame-shift^4^
Y127	Y22-3	*GSH1*	Glutathione biosynthesis	G839A	R280H
Y127	Y22-3	None	Subtelomeric Ty element	A317ins^5^	NA
Y128	Y127	*GRE3*	Aldose reductase	G136A^6^	A46T
Y128	Y127	*IRA2*	Inhibitor of RAS	G8782T	E2928Stop
Y128	Y127	*SAP190*	Component of Sit4p phosphatase complex	A2590G	S864G

^1^ *Saccharomyces* Genome Database (http://www.yeastgenome.org/).

^2^ Nucleotide and position in parent to evolved mutation.

^3^ Amino acid and position in parent to evolved amino acid.

^4^ Deletion mutation caused a codon shift in the reading frame.

^5^ Insertion of A occurs after nucleotide position 317 in the telomeric region of the left arm of Chromosome XIV.

^6^ Published in [10].

During directed evolution, random mutations with neutral or minimal impact on selective growth (so called “hitchhiker” mutations; [35]) can be carried along with beneficial “driver” mutations. Thus, to define the contributions of the *hog1*, *isu1* and *gsh1* mutations for aerobic xylose metabolism by Y127, we backcrossed Y127 of opposite mating type to the Y22-3 parent. Forty individual haploid progeny from ten tetrads were then genotyped and phenotyped for their aerobic xylose consumption rates per unit cell mass in comparison to their parental strains. We then compared the genotyped progeny to the Y22-3 and Y127 parent strains (**Fig 1B**). Progeny containing only the *hog1* or *gsh1* mutation alone consumed xylose aerobically at similar levels to the non-xylose metabolizing Y22-3 parent, whereas strains harboring only the *isu1* mutation consumed xylose albeit at a slower rate compared to the evolved Y127 strain. In contrast, progeny containing both *isu1*^*H138Y*^ and *hog1*^*M282fs*^ mutations consumed xylose aerobically at significantly faster rates than the *isu1*^*H138Y*^ single mutant and similarly to both the *isu1*^*H138Y*^ *hog1*^*M282fs*^ *gsh1*^*R280H*^ triple mutant progeny and the evolved Y127 parent. We conclude that the mutation in *ISU1* was required for the Y127 xylose metabolism phenotype and its effect was augmented by the *hog1*^*M282fs*^ mutation for the Y127 aerobic xylose metabolism phenotype.

### Additional mutations in *GRE3* and *IRA2* contribute to anaerobic xylose fermentation

We next identified the mutations responsible for anaerobic xylose fermentation by the evolved Y128 strain. From sequence comparisons between Y22-3 and Y128, we identified the *isu1*^*H138Y*^, *hog1*^*M282fs*^, and *gsh1*^*R280H*^ mutations present in Y127 and three additional Y128-specific mutations: (i) the missense mutation in *GRE3* reported earlier [10]; (ii) a non-synonymous SNP in *IRA2*, which encodes a negative regulator of Ras and is an inhibitor of cAMP-PKA signaling [36]; and (iii) a non-synonymous SNP in *SAP190*, which encodes a component of the Sit4p phosphatase complex [37] and is involved in TOR signaling [38] (**Table 1**). The mutation in *IRA2* causes a nonsense coding change that removes 152 carboxy-terminal amino acids, a region important for Ira2p stability [39]. Loss of *IRA2* function is known to activate Ras, subsequently stimulating PKA kinase activity on various target proteins, including trehalose biosynthesis, glycolytic enzymes, and transcription factors controlling ribosomal protein expression and stress response [16]. The missense mutation in *SAP190* causes a serine 864 to glycine change. We next crossed the Y128 strain with the Y127 strain of opposite mating type and generated 7 tetrads and 28 haploid progeny, all of which had the *isu1*^*H138Y*^, *hog1*^*M282fs*^ and *gsh1*^*R280H*^ mutations common to both Y127 and Y128. These haploid progeny were then genotyped and phenotyped for their rates of anaerobic xylose consumption per unit cell biomass (**Fig 1C**) in comparison to Y128 and its predecessor Y127, which does not consume xylose anaerobically. Descendants with either the single *ira2*^*E2928Stop*^ or *gre3*^*A46T*^ mutations, in the context of *isu1*^*H138Y*^ and *hog1*^*M282fs*^ mutations present in Y127, consumed xylose faster than Y127, but slower than Y128. In contrast, double *ira2*^*E2928Stop*^ *gre3*^*A46T*^ and triple *ira2*^*E2928Stop*^ *gre3*^*A46T*^ *sap190*^*S864G*^ mutants (also harboring the Y127 mutations), fermented xylose at rates equivalent to Y128 and significantly faster than *gre3*^*A46T*^ single mutants. Progeny containing the *sap190*^*S864G*^ mutation in combination with an *ira2*^*E2928Stop*^ or *gre3*^*A46T*^ mutation fermented similar amounts of xylose as single *ira2*^*E2928Stop*^ or *gre3*^*A46T*^ mutant strains.

### Mutations in *ISU1* and *HOG1* interact epistatically for rapid aerobic xylose consumption

**Fig 2:**
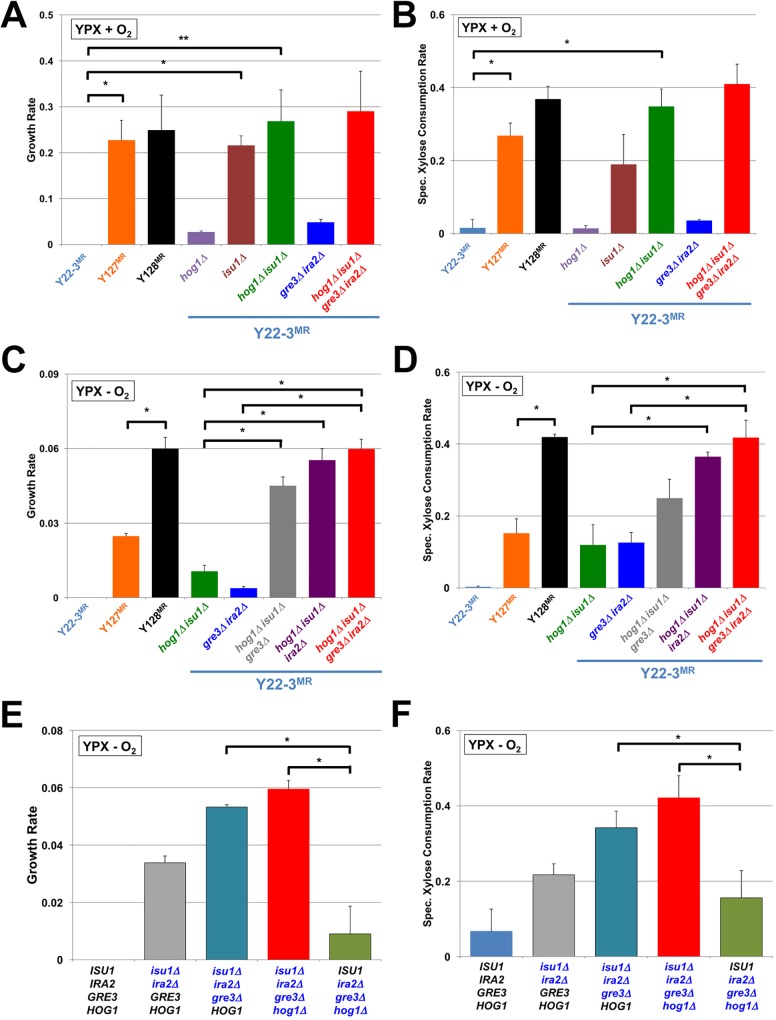
Deletions of *ISU1*, *HOG1*, *GRE3* and *IRA2* are sufficient to increase cell growth and xylose consumption rates. Indicated strains were cultured in YPX media under aerobic (**A-B**) or anaerobic (**C-F**) conditions. Average growth and specific xylose consumption rates with standard deviations are reported in g of dry cell mass•hr^-1^ (**A, C**) or OD600•hr^-^1 (**E**), and g of xylose consumed•g of dry cell mass^-1^•h^-1^ (**B, D**) or g of xylose consumed•OD_600_^-1^•h^-1^ (**F**), respectively, from the indicated strains cultured in YPX media from three independent biological replicates. Asterisks denote statistically significant differences (*; *P* < 0.05, **; *P* < 0.061) between specified strains by paired Student’s t-test. Xylose consumption rates for all strains in (**D**) were significantly faster (*P* < 0.05) than Y22-3^MR^.

Given that the biological functions of *HOG1*, *ISU1*, and *IRA2* have not been previously connected to xylose metabolism (**Fig 1D**), we sought to validate the requirement for the mutations in these genes in xylose metabolism by introducing targeted deletion mutations in a derivative of the Y22-3 parent strain that lacked the *kanMX* antibiotic marker used to integrate the *XylA-XYL3-TAL1* expression cassette (Y22-3^MR^, MR, marker-rescued). We first attempted to reconstruct the Y127 aerobic xylose metabolism phenotype by deleting *HOG1* and *ISU1*. Y22-3^MR^ strains harboring various combinations of *hog1Δ, ira2Δ* and *gre3Δ* mutations were generated and examined for their abilities to grow on and consume xylose aerobically as the sole sugar source (**Fig 2A and 2B**). We calculated cell growth and specific xylose consumption rates (xylose consumed per unit cell mass), which corrected for differences in xylose consumption due to variation in culture densities, and found that, not surprisingly, the relative differences in growth and consumption rates closely correlated with each other (confirming that growth is dependent upon xylose consumption). The single *isu1Δ* mutant aerobically grew on and consumed xylose faster than the wild-type Y22-3^MR^ parent. In contrast, single-gene deletion of *HOG1* had no effect. However, deletion of *HOG1* in the context of the *isu1Δ* mutation significantly increased the aerobic growth and xylose consumption rates compared to the *isu1Δ* mutation alone, with rates equivalent to the Y127 marker-rescued strain (Y127^MR^), revealing an epistatic interaction between the two mutations. Deletion of *GSH1* alone or in combination with other mutations (**S1A and S1B Fig**) did not produce statistically significant differences in xylose consumption rates compared to *isu1Δ* and *hog1Δ isu1Δ* strains, confirming that *GSH1* deletion contributed little to the xylose metabolism phenotype. Additionally, strains engineered with deletion mutations in the paralog *ISU2* did not consume xylose faster than Y22-3^MR^ (**S1C and S1D Fig**). Together, these results indicate that synthetic genetic interactions between *hog1Δ* and *isu1Δ* mutations enable rapid aerobic growth on and consumption of xylose.

### Deletion of *IRA2*, *GRE3*, *HOG1*, and *ISU1* enables rapid anaerobic xylose fermentation

We previously reported that deletion of *GRE3* in the Y127^MR^ *gre3Δ* mutant strain enabled faster anaerobic xylose fermentation than in Y127^MR^ but not at the same rate as Y128^MR^ [10]. Given the identification of the *ira2*^*E2928Stop*^ mutation in Y128, we next assessed whether specific deletion of *IRA2* could increase the rate of anaerobic xylose consumption comparable to Y128^MR^. Indeed, deleting *GRE3* and *IRA2* in the evolved Y127^MR^ and Y128^MR^ genetic backgrounds enabled cells to consume and grow on xylose anaerobically at rates equivalent to Y128^MR^ (**S2A and S2B Fig**). Additional deletion of *SAP190* had no effect in the Y127^MR^ *gre3Δ ira2Δ* background but impaired xylose consumption and growth in the Y128^MR^ *gre3Δ ira2Δ* background (**S2A and S2B Fig**). Interestingly, deletion of the *IRA2* paralog, *IRA1*, in the Y127^MR^ *gre3Δ* background yielded a strain with intermediate rates of anaerobic xylose consumption and growth compared to Y127^MR^ *gre3Δ* and Y127^MR^ *gre3Δ ira2Δ* mutants (**S2C and S2D Fig**). We conclude that loss-of-function mutations in *IRA2* contribute to anaerobic xylose consumption and that alternative loss of *IRA1* function can also facilitate moderate anaerobic xylose fermentation, indicating that Ira2p and Ira1p are not entirely redundant for function. These observations could reflect differences in either activities or expression levels.

As a further test for the role of *ira2* and *gre3* mutations in anaerobic xylose fermentation, we determined the cell growth, specific xylose consumption and ethanol production rates of Y22-3^MR^ strains engineered with various combinations of deletions in flasks (**Fig 2C and 2D, S3A Fig**) and in controlled bioreactors sparged continuously with N_2_ gas (**S4 Fig**). Deletion of *IRA2* or *GRE3* increased the growth, specific anaerobic xylose consumption, and ethanol production rates in the context of the *hog1Δ isu1Δ* double knockout. Moreover, simultaneous deletion of *HOG1*, *ISU1*, *IRA2*, and *GRE3* resulted in specific xylose consumption and ethanol production rates comparable to Y128^MR^. Interestingly, double deletion of *GRE3* and *IRA2* alone had limited impacts on aerobic (**Fig 2A and 2B**) or anaerobic (**Fig 2C and 2D, S3A and S4 Figs**) xylose consumption, ethanol production and growth relative to the Y22-3^MR^ parent, suggesting that loss-of-function *hog1* and *isu1* mutations were crucial for enabling anaerobic xylose fermentation. Indeed, we found that deletions of *ISU1*, *GRE3*, and *IRA2* together conferred anaerobic growth, consumption, and ethanol production on xylose nearly equivalent to deletion of all four genes (**Fig 2E and 2F, S3B Fig**), but deletions of *HOG1*, *GRE3*, and *IRA2* had a minimal effect. Together, these results indicate that loss of *ISU1* function is a major contributor to anaerobic conversion of xylose.

To determine the generality of the effects caused by mutations in these newly-implicated pathways, we engineered xylose catabolism into two different, commonly used laboratory yeast strains: BY4741 [40], which was derived from S288c, and CEN.PK113-5D [41], a derivative of CEN.PK2 that is often engineered for xylose metabolism studies [42]. BY4741 and CEN.PK113-5D strains were engineered with the same DNA cassette that allowed expression of bacterial xylose isomerase, fungal *XYL3* and yeast *TAL1* [10] in the Y22-3^MR^ strain. Subsequent deletions of *HOG1*, *ISU1*, *GRE3*, and *IRA2* were sufficient to confer significantly faster anaerobic consumption of xylose in both BY4741 and CEN.PK113-5D backgrounds (**S5B Fig**), and significantly faster cell growth and ethanol production in the CEN.PK113-5D background (**S5A and S5C Fig**). Thus, the combined abilities of the *hog1Δ*, *isu1Δ*, *gre3Δ*, and *ira2Δ* mutations to confer anaerobic conversion of xylose into ethanol are not limited to the Y22-3^MR^ strain background.

### Mutations enabling xylose metabolism also affect metabolism of other carbon substrates

**Fig 3:**
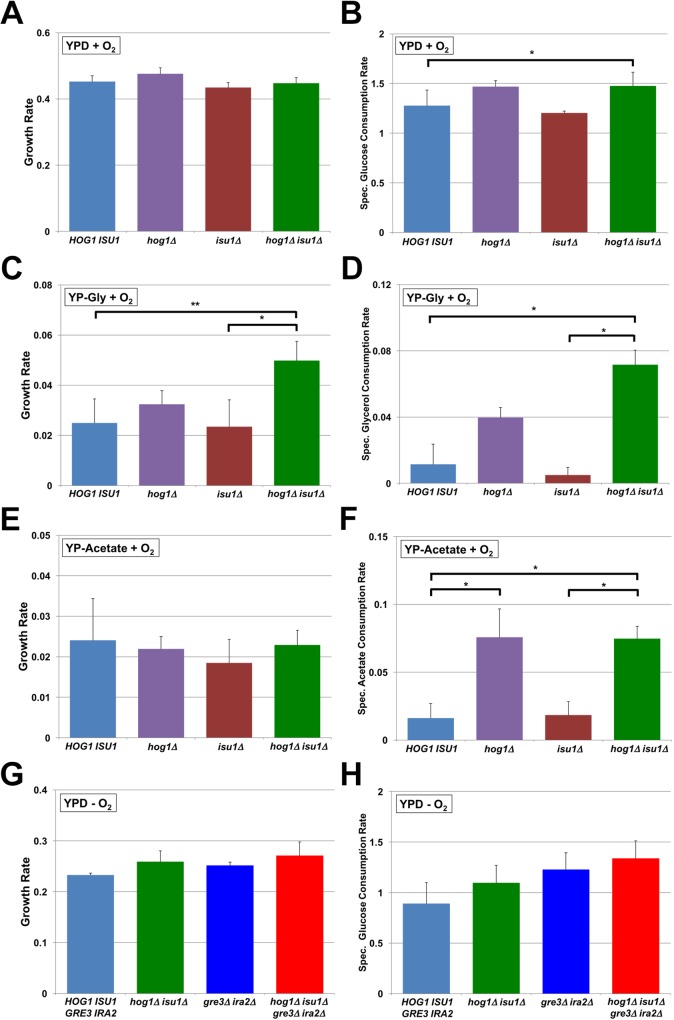
Deletions of *HOG1* and *ISU1* have different effects on the metabolism of other carbon substrates. Graphs display growth and consumption rates for Y22-3^MR^ strains containing the indicated genotypes cultured aerobically in YP media containing glucose (**A-B**), glycerol (**C-D**), acetate (**E-F**), or anaerobically with glucose (**G-H**). Reported values are averages and standard deviations from biological triplicate experiments, and in g substrate consumed or ethanol produced•L^-1^•h^-1^•cell mass (in OD_600_)^-1^. Asterisks denote statistically significant differences (*, *P* < 0.05; **, *P* < 0.068) by Student’s t-test.

Although we carried out directed evolution specifically on xylose, the roles of Hog1p, Isu1p, and Ira2p in biochemical pathways of broad function raised the possibility that these evolved mutations could impact carbon metabolism more generally. We tested this possibility by measuring the growth and consumption rates of various deletion strains on a variety of carbon sources, which are consumed through different entry points of central metabolism compared to xylose (**S6A Fig**). For glucose, the mutations had minimal effects on aerobic growth and consumption rates (**Fig 3A and 3B**). In contrast, we found that *hog1Δ isu1Δ* mutants grew on and consumed glycerol (**Fig 3C and 3D**) and acetate (**Fig 3E and 3F**) significantly faster than the parental Y22-3^MR^ strain under aerobic conditions. On the other hand, *hog1Δ* and *isu1Δ* single mutations caused modest or no increases in galactose (**S6B and S6C Fig**) and ethanol (**S6D and S6E Fig**) growth and consumption rates. Unlike the effect of *isu1Δ* on xylose consumption, deletion of *ISU1* did not improve the consumption rates of these other carbon substrates significantly. Rather, deletion of *HOG1* alone, which had no effect on aerobic xylose metabolism, resulted in significantly faster glycerol and acetate consumption rates with slight to no effect on growth rates. Quadruple deletions of *HOG1*, *ISU1*, *GRE3* and *IRA2* did not significantly alter anaerobic growth or glucose consumption rates (**Fig 3G and 3H**), but produced significantly faster growth on and consumption of galactose anaerobically than other combinations of the mutations (**S6F and S6G Fig**). This suggests that the combined mutations enabling xylose metabolism also confer more rapid consumption of non-preferred carbon substrates, but that the genetic architectures and epistatic interactions vary for each carbon source.

### Proteomic analysis revealed altered abundance of proteins involved in metabolism and stress response

**Fig 4:**
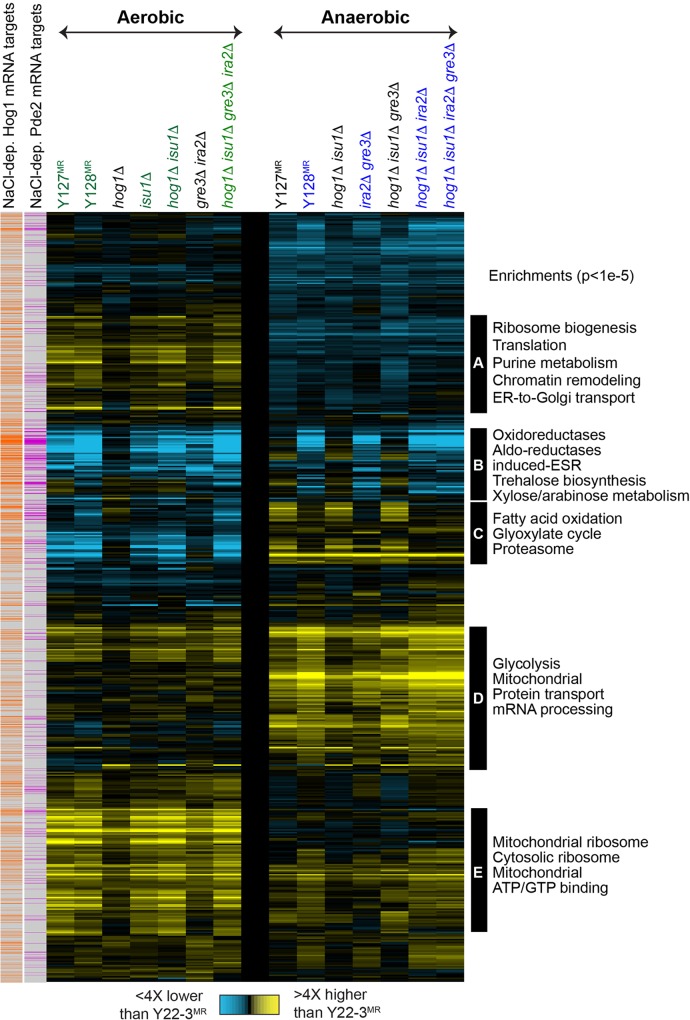
Proteomic changes across strains implicate physiological responses. The heat map shows the average relative protein abundance for 3,660 proteins (rows) in each denoted strain (columns) compared to Y22-3^MR^ grown aerobically (left) or anaerobically (right) on xylose as the sole carbon source. Genotypes indicated in green or blue denote strains that can grow on xylose aerobically or contain mutations in *IRA2*, respectively. Yellow indicates higher abundance and blue indicates lower abundance according to the key. Data were pooled and organized by hierarchical clustering [95]. Functional groups enriched in denoted clusters at p < 1e-5 (hypergeometric test [94]) are annotated to the right of each cluster. Proteins encoded by mRNAs whose salt-dependent expression is dependent on Hog1 or the phosphodiesterase Pde2 (which indirectly represses PKA activity through cAMP degradation, [21]) as defined in [48] are shown as orange or magenta boxes, respectively.

To shed light on the molecular mechanisms by which the mutations in *HOG1*, *ISU1*, and *IRA2* increased the rate of xylose metabolism by yeast, we compared the protein abundances of the various strains normalized to the proteome of the parental Y22-3^MR^ strain cultured in xylose aerobically and anaerobically. We identified proteins and enriched functional groups whose abundances were significantly different across strains by statistical (False discovery rate, FDR <0.05, **S7A Fig, S3 and S4 Appendixes**) and clustering (**Fig 4**) analyses. We first compared proteome differences in Y127^MR^ and the parental Y22-3^MR^ strains grown aerobically on xylose as the sole carbon source (**S3 and S4 Appendixes**); over 800 proteins showed statistically significant abundance differences across the two strains. Proteins at lower abundance in the evolved Y127^MR^ strain versus the parental Y22-3^MR^ strain were enriched for proteins involved in alternative energy usage, stress defense, including proteins linked to alternative carbon metabolism, ethanol catabolism, fatty acid β-oxidation, the pentose phosphate shunt, and proteins in the environmental stress response (ESR, [43], **Fig 4**, Clusters B and C). These differences are consistent with a starvation response in Y22-3^MR^ that has been alleviated in the evolved Y127^MR^ strain. Altered starvation responses have been linked to differences in growth rate [44–46], which could also impact their relative abundances in Y127^MR^ versus Y22-3^MR^ strains (see below for additional discussion). Additionally, Y127^MR^ displayed higher abundance of approximately 72% of 1,208 mitochondrial proteins reported elsewhere ([47], **Fig 4**, Clusters D and E). The proteins in these clusters included those linked to mitochondrial transport, translation, and respiration, as well as proteins involved in lipid biogenesis and Golgi and ER functions (**S7A Fig**). Deletion of *ISU1* alone, which caused an intermediate enhancement in xylose consumption rate, recapitulated many of the proteomic differences seen in Y127^MR^, including increased the abundance of mitochondrial proteins (**Fig 4**, Cluster D and E) that may have enabled faster xylose consumption rates. In contrast, deletion of *HOG1* alone caused minor differences in protein abundances compared to Y22-3^MR^. Interestingly, deletion of *HOG1* in the context of the *ISU1* deletion did not significantly alter mitochondrial protein abundances relative to the *isu1Δ* mutant. This strongly suggests that the increased abundances of mitochondrial proteins in **Fig 4,** Clusters D and E were responses to the *isu1* mutations.

We next compared the proteomes of strains with varying abilities to ferment xylose anaerobically (**Fig 4**, **S7B Fig**, **S3**and **S5 Appendixes**). Both Y128^MR^ and quadruple deletion mutants displayed signatures of elevated PKA signaling compared to Y127^MR^ and *hog1Δisu1Δ* double mutants, including lower abundance of proteins linked to stress defense and trehalose biogenesis (**Fig 4**, Cluster B), and higher abundances of proteins involved in glycolysis (**Fig 4**, Cluster D) and ribosome biogenesis (**Fig 4**, Cluster E). Indeed, many of the mRNA targets in these clusters are regulated by the phosphodiesterase Pde2, which also inhibits PKA signaling similar to Ira2, as well as Hog1, in response to salt stress [48]. Strains containing *ira2Δ* or *ira2*^*E2928Stop*^ mutations specifically displayed both lower expression of trehalose biosynthesis enzymes and intracellular trehalose levels (**S8 Fig**), which have been observed elsewhere [49, 50]. Unexpectedly, *isu1Δ* and *isu1*^*H138Y*^ mutant strains also contained lower intracellular trehalose under aerobic conditions (**S8D Fig**). Some of these proteomic changes could be simply a secondary response from strains capable of growing on xylose versus Y22-3^MR^, which cannot. However, at least some are likely to be a direct regulatory response to the mutations. For example, deletion of *IRA2* produced many of the growth-related signatures (*i*.*e*., lower abundance of stress proteins and higher abundance of many proteins related to ribosome biogenesis), even though this strain cannot grow on xylose without additional mutations (see Fig **2A**). This result shows that regulation of the growth-correlated signature is a direct response of PKA signaling, which can be decoupled from growth. Unlike the other mutations, deletion of *GRE3* did not produce major proteome changes even though it significantly enhanced xylose fermentation and cellular growth rate on xylose. This suggests that the main contribution of *gre3* mutations is to minimize xylitol production, and further indicates that differences in growth rate cannot explain the proteome differences we observe in these conditions.

### Xylose-metabolizing strains exhibited altered pentose phosphate pathway activities compared to non-xylose metabolizing strains

**Fig 5:**
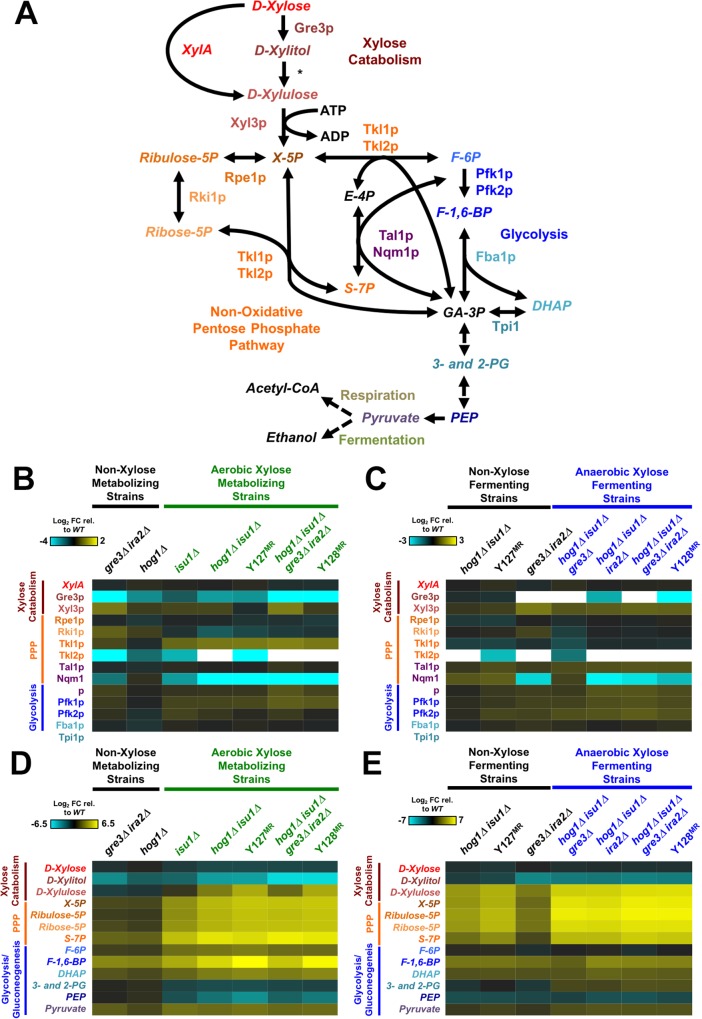
Xylose-metabolizing strains have altered pentose phosphate pathways. Heat maps display intracellular concentrations of proteins and metabolites from xylose metabolism, pentose phosphate pathway and glycolysis (**A**) from engineered and evolved strains relative to Y22-3^MR^. Colors correspond to average Log_2_ fold change values from strains cultured under aerobic (**B** and **D**) or anaerobic (**C** and **E**) conditions in YPX medium from three biological replicates. White boxes indicate strains for which no corresponding peptides were detected. The asterisk (*) in (**A**) denotes an enzyme activity that is absent in the Y22-3^MR^ strain. Abbreviations: X-5P, xylulose-5-phosphate; S-7P, sedoheptulose-7-phosphate; F-6P, fructose-6-phosphate; F-1,6-BP, fructose-1,6-bisphosphate; DHAP, dihydroxyacetone-phosphate; 3- and 2-PG, 3- and 2-phosphoglycerates; PEP, phosphoenolpyruvate.

Based on the proteome-scale signatures, we focused further comparative analyses on proteins and metabolites directly involved in xylose metabolism. Catabolism of xylose to ethanol in Y22-3^MR^ and evolved strains occurs by assimilation of xylose via native hexose transporters Gal2p, Hxt4p, Hxt5p and Hxt6/7p [51], xylose conversion to xylulose-5-phosphate, which is then metabolized through the non-oxidative pentose phosphate and glycolytic pathways into ethanol anaerobically or respired aerobically (**Fig 5A**). As expected, Y128^MR^ and strains containing *gre3Δ* mutations expressed very low or undetectable levels of Gre3p peptides relative to Y22-3^MR^, suggesting that the *gre3*^*A46T*^ mutation may destabilize the protein (**Fig 5B and 5C**). Strains with mutations in *HOG1* also had lower abundances of Gre3p compared to Y22-3^MR^, which is consistent with a report that Hog1p regulates *GRE3* expression [52]. Interestingly, under both aerobic and anaerobic conditions, we observed relatively low or undetectable levels of Tkl2p in Y127^MR^ and *isu1Δ* strains; Tkl2p is one of two trans-ketolases functioning in the pentose phosphate pathway. We also observed low levels of Nqm1p, a paralog of transaldolase Tal1p with unknown function, in all xylose-metabolizing strains relative to the Y22-3^MR^ parent. The metabolomic profile of the same strains indicated that both aerobic and anaerobic xylose-fermenting strains accumulated more pentose phosphate intermediates than Y22-3^MR^ (xylulose-5-phosphate, ribulose-5-phosphate, ribose-5-phosphate and sedoheptulose-7-phosphates; **Fig 5D and 5E**). These patterns suggest that the combinations of enabling mutations in Y127 and Y128 overcame several of the initial bottlenecks in import and conversion of xylose into xylulose-5-phosphate and thereby uncovered downstream bottlenecks in the pentose phosphate pathway and beyond, increasing the levels of pentose phosphate intermediates. In contrast, there was no strong correlation between TCA cycle enzyme abundance and strain phenotypes aerobically or anaerobically (**S9 Fig**), although significant changes in TCA cycle metabolites appeared in aerobic xylose metabolizing strains. Specifically, aerobic xylose-metabolizing strains accumulated higher levels of succinate and lower levels of citrate, aconitate, isocitrate, and malate relative to non-xylose metabolizing strains (**S9D and S9E Fig**). However, these differences could be due to the fact that these metabolites are shared with the glyoxylate cycle (see below).

### *Isu1Δ* mutation has essential aerobic and anaerobic functions in xylose metabolism

**Fig 6:**
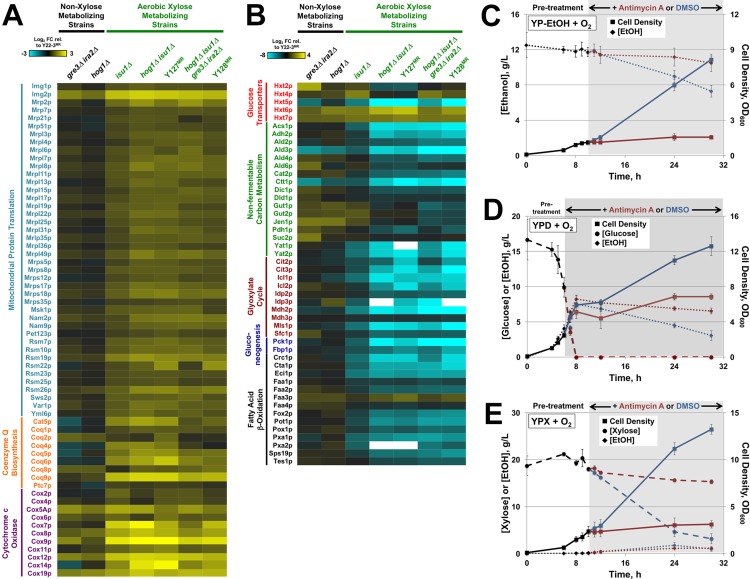
Mutations in *ISU1* enhance respiration of xylose. Engineered and evolved strains were cultured in aerobic YPX media and analyzed for intracellular protein and metabolite concentrations. Average Log_2_ intracellular concentrations of mitochondrial translation and respiration proteins (**A**) or hexose transporters and glucose-repressed proteins (**B**) from mutant strains relative to the Y22-3^MR^ parent are shown. White boxes indicate strains for which no corresponding peptides were detected. Relative protein concentrations were calculated from three independent biological replicates. Y22-3^MR^ *hog1Δ isu1Δ* strains were cultured in YP-Ethanol (**C**), YPD (**D**) or YPX (**E**) media and then treated with DMSO control or 0.5 μg/mL Antimycin A. Shaded areas represent the time during which DMSO or Antimycin A were present in the cultures. Average cell density, sugar and ethanol concentration with standard deviations from three independent biological replicates are reported.

Pair-wise proteomic comparisons between strains with *isu1Δ* or *isu1*^*H138Y*^ mutations to strains with wild-type *ISU1* identified increased abundance of proteins functioning in mitochondrial protein translation and respiration, and decreased abundance of glucose repressed proteins under aerobic conditions (**Fig 4 and S7A Fig**). We next directly compared the abundances of proteins involved in mitochondrial translation, coenzyme Q biosynthesis [53] and cytochrome c oxidase subunits [54] in all strains according to their abilities to grow on and metabolize xylose aerobically (**Fig 6A**). Noticeably, most of these proteins were expressed at higher abundances in all five xylose-metabolizing strains with the *isu1Δ* or *isu1*^*H138Y*^ mutations compared to strains with wild-type *ISU1*. Conversely, glucose-repressed proteins involved in the metabolism of non-fermentable carbon substrates (*e*.*g*., glycerol, ethanol, and acetate), in the glyoxylate cycle, in gluconeogenesis, and in fatty acid β-oxidation were expressed at lower abundances in aerobic xylose-metabolizing strains compared to non-xylose metabolizing strains (**Fig 6B**). Interestingly, yeast lacking the Yeast Frataxin Homologue 1 (*YFH1*), which functions together with Isu1p in Fe-S cluster assembly, down regulated expression of the same glucose-repressed genes when grown on glycerol [55]. The hexose transporter Hxt5p, which is induced in the presence of non-fermentable carbon sources [56], was similarly detected at lower levels in xylose-metabolizing strains. These results also indicated that altered succinate, citrate, acontitate, isocitrate, and malate levels between strains likely reflected changes in the concentrations of glyoxylate cycle intermediates (**S9B Fig**). Similar proteomic differences among xylose-fermenting and non-fermenting strains were not seen under anaerobic conditions (**S10 Fig**), indicating that these effects were specific to aerobic conditions. These proteomic patterns suggest that strains containing *isu1Δ* or *isu1*^*H138Y*^ mutations were not starved on xylose aerobically and did not activate pathways that scavenge non-fermentable carbon sources from the medium, whereas strains with wild-type *ISU1* equated the presence of xylose as the sole carbon source to glucose depletion.

The capacity to respire xylose could explain the emergence of the *isu1*^H138Y^ mutation in Y127 during the directed evolution of Y22-3 on xylose aerobically. To determine whether xylose was respired under aerobic conditions, we treated *hog1Δ isu1Δ* mutant cells grown with Antimycin A, an inhibitor of oxidative phosphorylation [57]. As expected, the addition of Antimycin A to medium containing ethanol, which can only be respired, blocked cell growth and ethanol consumption one h after treatment, whereas the DMSO only-treated culture continued to growth on and consume ethanol (**Fig 6C**). We next treated the same yeast strain cultured aerobically on glucose, which can be fermented into ethanol in the presence of oxygen. In contrast, the *hog1Δ isu1Δ* mutant strain continued to grow on and ferment glucose into ethanol up to 2 h after Antimycin A treatment (**Fig 6D**), indicating that Antimycin A did not affect fermentative growth and metabolism. Once all of the glucose was consumed by 8 h, the culture grew from respiration of ethanol with DMSO treatment but not with Antimycin A. Treatment with Antimycin A, but not with DMSO, profoundly blocked aerobic growth on and consumption of xylose similar to what was seen for ethanol (**Fig 6E**). Together, these results indicate that loss of function mutations in *ISU1* and *HOG1* enabled growth on and catabolism of xylose through respiration. This result was surprising given that the *isu1Δ* mutations are required for anaerobic xylose fermentation, revealing an oxygen-independent role for *ISU1* mutations in driving xylose metabolism.

## Discussion

**Fig 7:**
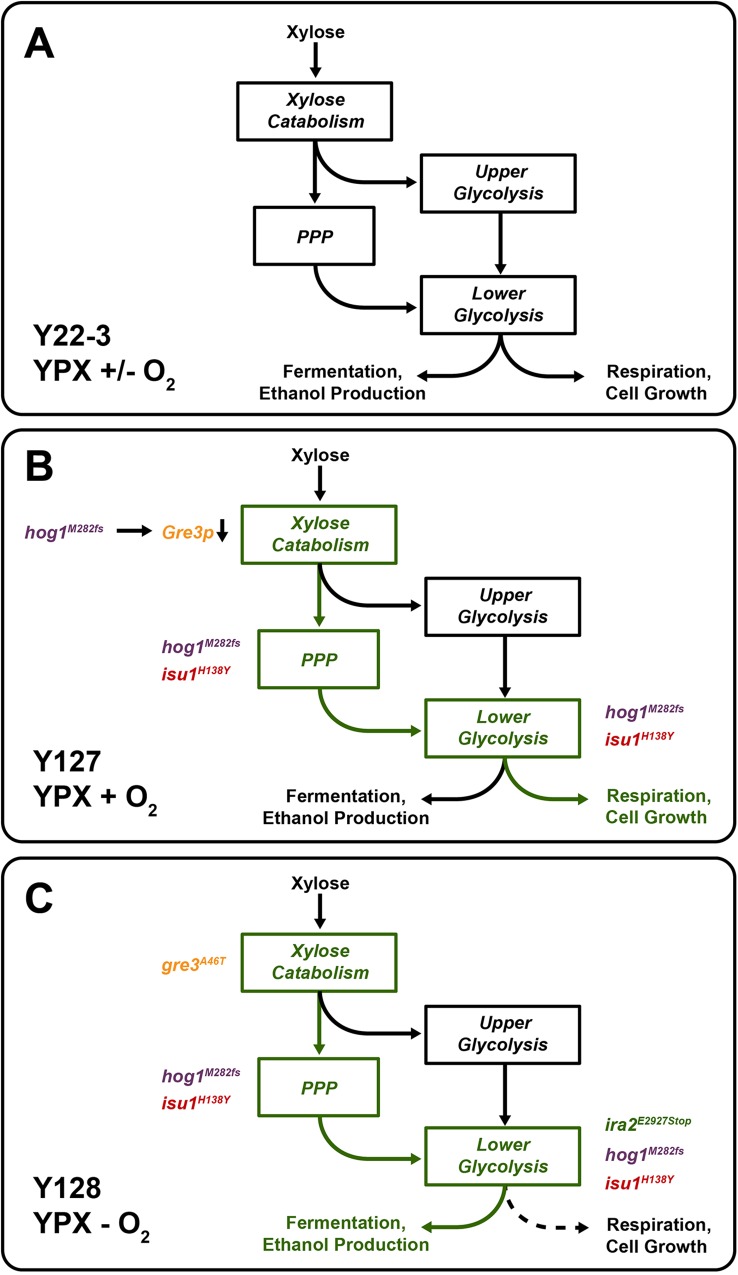
Proposed model for how the evolved mutations impact biochemical pathways for xylose metabolism. Text, shapes and arrows in green signify upregulated activities compared to the activities in the parental strain (in black). Under aerobic or anaerobic conditions, the parent strain consumes low amounts of xylose due to insufficient biochemical activities (**A**). Under aerobic conditions, the evolved *hog1*^*M282fs*^ and *isu1*^*H138Y*^ mutations enhance activities (signified in green) of the pentose phosphate and lower glycolytic pathways, as well as respiration, thereby permitting significantly greater growth on and metabolism of xylose (**B**). Loss of *HOG1* function caused reduced expression of *GRE3* and other targets that impair xylose metabolism. Under anaerobic conditions (**C**), the evolved *ira2*^*E2928Stop*^ mutation causes activation of PKA, which in turn activates glycolytic enzymes. This, along with the disabling *gre3*^*A46T*^ mutation, enables the fermentation of xylose into ethanol.

Through combined genome sequence comparisons and genetic approaches, we identified novel epistatic genetic interactions between mutations in *HOG1*, *ISU1*, *GRE3*, and *IRA2* that enabled anaerobic xylose fermentation across multiple yeast strains engineered with xylose isomerase. Mutations in *GRE3* and *IRA2* were only beneficial for anaerobic xylose fermentation and required additional mutations in *HOG1* and *ISU1* to fully recapitulate the evolved Y128 phenotype (**Fig 2C and 2D**). Based on our combined genetic, proteomic, and metabolomic studies, we propose a model by which the mutations in the evolved Y127 and Y128 strains emerged and enabled xylose metabolism (**Fig 7**). The parental Y22-3 strain lacks sufficient metabolic activities in one or more steps in xylose catabolism, the pentose phosphate pathway, the glycolytic pathway, or some combination, to permit significant growth and fermentation of xylose aerobically or anaerobically (**Fig 7A**). Due to this inability, Y22-3 and other non-xylose metabolizing strains under aerobic conditions experience starvation stress, and respond by activating the ESR and Snf1p-controlled pathways that ordinarily allow metabolism of non-preferred and non-fermentable carbon substrates using the glyoxylate cycle (**Figs 4**and **6B**). Other transcriptomic and metabolomic studies of xylose metabolism identified up-regulation of glyoxylate cycle and non-fermentable carbon metabolism genes [58–62]; however, in some cases, these data were interpreted as an indication that up-regulation of these pathways was needed for xylose metabolism.

Despite the inabilities of Y22-3 to metabolize xylose, the power of aerobic selective pressure drove the emergence of the Y127 strain. The acquisition of mutations in *HOG1* and *ISU1* permitted the Y127 strain to overcome a number of biochemical bottlenecks for aerobic xylose metabolism (**Fig 7B**), possibly through altered xylose catabolism and pentose phosphate pathway activities (**Fig 5B and 5D**). Loss of *ISU1* function also enhanced aerobic respiration of xylose, as suggested by up-regulation of respiratory proteins in *isu1* mutants (**Fig 6A**) and the complete block in aerobic xylose consumption by Antimycin A (**Fig 6C–6E**). Previous studies indicate that mitochondrial protein translation and oxidative phosphorylation are tightly co-regulated (reviewed in [63]). Specifically, Fe-containing heme is involved in both translation and assembly of cytochrome c oxidase (COX) subunits into the final enzymatic complex of the mitochondrial respiration chain [64, 65]. Thus, loss of *ISU1* function may have impaired Fe-S cluster formation and increased the availability of Fe for heme biosynthesis. This could promote the formation of active COX complexes to enhance respiration of xylose and other carbon substrates (**Figs 2 and 3**, **S6 Fig**). Alternatively, loss of *ISU1* function may have caused an expansion in mitochondrial mass to compensate for the reduced capacity to generate Fe-S clusters. Either case could cause increased abundances of mitochondrial proteins in *isu1* mutants (**Figs 4**and **6A**) and subsequently result in faster xylose respiration. However, the *isu1* mutations were also required for anaerobic xylose growth, which involves fermentation and part of glycolysis but not respiration; thus, the role of *isu1* mutations in anaerobic xylose use is unclear and will require further investigation.

The mechanistic explanation for the emergence of mutations in *HOG1* may be related to its suppression of metabolism and growth during stress through phosphorylation of transcriptional regulators or cytosolic factors. First, we (**Fig 5B and 5C**) and others [12] found that Hog1p positively regulates the expression of *GRE3*. Thus, the lowered expression of Gre3p in *HOG1* mutants reduced the production of inhibitory xylitol and permitted faster xylose catabolism. Additionally, both limited glucose availability [66] and non-fermentable carbon substrates [67] were found to activate Hog1p. Snf1p, which is essential for metabolism of non-preferred carbon sources, also plays a critical role in regulating Hog1p activity under endoplasmic reticulum and starvation stress [67, 68]. The role of Hog1 activation under these conditions was thought to decrease production of cellular biomass, perhaps to balance growth demand with energy availability. Although it is certainly possible that Hog1 mediates transcriptional responses that normally inhibit xylose metabolism, Hog1-mediated phosphorylation of metabolic enzymes, such as GAPDH/Tdh3 [69], may also limit xylose utilization. Thus, we propose that deletion of *HOG1* relieves growth inhibition and restores glycolytic activity in response to non-glucose carbon sources. With enhanced respiration providing greater energy yield from xylose with *isu1* mutations, the additional mutations in *HOG1* subsequently allow for uninhibited aerobic growth on xylose.

The specific impacts of mutations in *GRE3* and *IRA2* in enabling anaerobic xylose metabolism, as well as their epistatic interactions with *hog1* and *isu1* mutations, were unexpected (**Fig 7C**). Gre3p is a known inhibitor of xylose isomerase through its production of xylitol from xylose, and deletion of *GRE3* is known to improve xylose metabolism [10, 13]. Nevertheless, the emergence of the *gre3*^*A46T*^ mutation during anaerobic evolution on xylose is notable, indicating that reduced *GRE3* expression from the *hog1*^*M282fs*^ mutation during aerobic evolution was insufficient. The cAMP-PKA pathway is a well-known positive regulator of sugar metabolism and cellular growth (reviewed in [16, 20, 70]); PKA phosphorylates and positively regulates the activities of key glycolytic enzymes, including pyruvate kinase (Cdc19 and Pyk2), for glucose fermentation. Thus, activation of PKA from the inactivating E2928 to stop mutation in *IRA2* may enhance glycolytic flux under anaerobiosis, resulting in faster conversion of xylose into ethanol (**Fig 7C**). Although other experimental evolution studies also uncovered mutations in *IRA* that provided fitness advantages under aerobic glucose limitation [35, 71], our studies identified a novel, anaerobic-specific function for PKA signaling since *ira2* mutations have little effect on aerobic xylose metabolism (**Fig 2C and 2D**), and unexpectedly require mutations in *ISU1* for anaerobic xylose consumption (**Fig 2E and 2F**). To date, a direct anaerobic function for *ISU1* has not been identified. Together, epistatic genetic interactions between the *hog1*^*M282fs*^, *isu1*^*H138Y*^, *gre3*^*A46T*^ and *ira2*^*E2928Stop*^ mutations enhance the rate of anaerobic xylose fermentation through the pentose phosphate and glycolytic pathways.

The conversion of xylose into ethanol by yeast is a major bottleneck in lignocellulosic biofuel production [2, 4]. Efficient and rapid xylose fermentation is necessary for cost effective production of biofuels. A number of studies have genetically engineered *S*. *cerevisiae* for xylose metabolism, including targeted overexpression of pentose phosphate pathway enzymes, as well as the deletion of *GRE3*, *PHO13* and genes involved side product pathways [2, 7, 8, 72, 73]. To date, only one study has employed genome sequence comparisons to identify candidate sequence differences involved in evolved xylose metabolism phenotypes [8], which subsequently identified a mutation in *PHO13*. Our genetic and multiomic studies provide the first direct evidence that loss of function mutations in *HOG1*, *ISU1*, *GRE3*, and *IRA2* enable xylose fermentation through altered xylose catabolism, pentose phosphate pathway, glycolysis and respiration, and provide an advance in the identification of new genetic targets and pathways for improving xylose metabolism. Nonetheless, challenges remain in developing yeast strains capable of fermenting xylose from lignocellulosic hydrolysates, which are well known to contain microbial inhibitors and toxins that impact yeast xylose fermentation and induce stress responses [74–77]. This is particularly true for strains containing loss of *HOG*1 function [31] or an activated cAMP/PKA pathway [25], both of which are known to result in reduced stress tolerance. Future studies will be aimed at defining specific genes and pathways that enable rapid and efficient conversion of xylose into biofuels in the presence of these lignocellulose-derived inhibitors.

## Materials and Methods

### Media

Standard undefined yeast lab medium was prepared as previous described [78]. Briefly, liquid and plate-based medium contained 10 g/L yeast extract and 20 g/L peptone (YP), and various sugar concentrations (X = 20–30 g/L xylose, D = 20 g/L dextrose/glucose, Gal = 20 g/L galactose, Gly = 20 g/L glycerol, EtOH = 15 g/L ethanol, Acetate = 20 g/L acetate). For anaerobic bioreactor experiments, YPX media also contained 50 mM potassium phosphate, pH 5.5.

### Yeast strains

Yeast strains used in this study are described in **S1 Table**. Generation of Y22-3, Y127 and Y128 strains is described elsewhere [10]. The Y174 and Y176 strains were constructed in an identical manner to Y22-3 by integrating the *ScTAL1-CpxylA-SsXYL3-loxP-kanMX-loxP* cassette into the *HO* locus of BY4741 [40] and CEN.PK113-5D [79], respectively, followed by excision of the *kanMX* marker by Cre recombinase [80]. The Y127 MATα strain (GLBRCY156) was generated by diploidization of Y127 [81], sporulation, tetrad dissection and mating type identification [78]. For backcrossing, Y22-3 or Y128 was mated to Y156, subjected to sporulation and tetrad dissection. All tetrad spores were verified for 2:2 segregation of mating type. Haploid spores from 10 sets of Y22-3 X Y157 tetrads were generated, from which the *hog*^*M282fs*^, *isu1*^*H138Y*^, and *gsh*^*R280H*^ mutations segregated 2:2 in all progeny. Similarly, haploid spores from 7 sets of Y157 X Y128 tetrads were generated, from which *gre3*^*A46T*^, *ira2*^*E2928Stop*^, and *sap190*^*S864G*^ mutations segregated 2:2 in all progeny. Deletion of *GRE3*, *ISU1*, *ISU2*, *IRA1*, *IRA2*, *GSH1 and SAP190* were performed by integration of polymerase chain reaction (PCR) product generated from *LoxP-kanMX-LoxP* or *LoxP-hphMX-LoxP* plasmid templates [10, 80] and primers containing 40–60 bp of homology flanking the targeted gene. For deletion of *HOG1*, gDNA from a *hog1Δ*::*kanMX4* mutant strain [82] was used as the PCR template. PCR products were purified and transformed [83] into the appropriate strains. Cre recombinase-mediated excision of *LoxP*-flanked antibiotic markers was carried out as published elsewhere [80]. All strains were confirmed for gene deletion and antibiotic marker excision by PCR with independent outside flanking primers. Sanger sequencing of PCR products and DNA plasmids was performed by the University of Wisconsin-Madison Biotechnology Center.

### Genomic DNA sequence comparisons

For identification of genome sequence differences between strains, including single nucleotide polymorphisms (SNPs) and indels, 100 bp paired end Illumina short reads from Y127 and Y128 genomic DNA were generated as previously described [30]. First, paired-end reads were mapped to either the reference genome Y22-3 [30] or a variant of the S288c reference genome (NC_001133, version 64, [84]) in which the Y22-3 alleles identified by GATK [85] were replaced using Bowtie2 [86] with default settings. Y127 and Y128 SNPs and indels were further identified with GATK using base quality score recalibration, indel realignment and duplicate removal. Default parameters were used except -mbq 25 to reduce false-positive variant calls. Variants were then filtered using the following suggested GATK criteria: QD < 2, FS > 60, MQ < 40. The identified variants were substituted into the S288c reference genome, and to this 100-bp paired end reads from the evolved strains were mapped, followed by GATK analysis as above to define mutations in the evolved strains. Mutations were also identified using similar parameters by mapping to the assembled Y22-3 genome [30]. Non-synonymous mutations in each strain were verified by genomic DNA extraction (Masterpure Yeast DNA Purification Kit, Epicentre), PCR with gene-specific primers (Phusion DNA Polymerase, New England Biolabs), purification of PCR products (QIAquick PCR Purification Kit, Qiagen), and Sanger sequencing (University of Wisconsin-Madison Biotechnology Center). One SNP in *HPA3* (A-to-C in nucleotide 10 of the coding sequence causing a threonine 4 to proline change) in Y127 identified from the Illumina sequencing was not confirmed by Sanger sequencing. Further investigation determined that this mutation occurred during propagation of the strain for isolation of genomic DNA. Silent and intergenic mutations were not independently verified. All DNA sequencing reads have been deposited in the NCBI SRA under BioProject PRJNA279877.

### Cell culturing and phenotypic growth assays

Aerobic tube and anaerobic flask growth and sugar consumption assays were performed as previously described [10] with some modifications. In the combined cell growth, proteomic and metabolomic studies, which generated data described in **Figs 2–6**and **S7–S10 Figs**, yeast cells were grown in YPD media to log phase and then shifted into flasks containing 250 mL YPX media at a concentration of optical density at λ = 600 nm (OD_600_) = 0.3 for strains that could grow on xylose or OD_600_ = 0.6 for strains that do not grow on xylose. For anaerobic experiments, cells were shifted into YPX media that was placed 16 h prior in an anaerobic chamber (Coy) containing 10% H_2_, 10% CO_2_, and 80% N_2_ gases, and grown by stirring with a magnetic stir bar. Cell density, extracellular xylose and ethanol concentration measurements were taken at 0, 6.5, 8.5, 11, 13 and 17 h after inoculation for aerobic experiments, and 0, 8, 10, 14, 19, 20, and 32 h after inoculation for anaerobic experiments. Cells were harvested 14 h (for aerobic cultures) or 20 h (for anaerobic cultures) for proteomic (see below), metabolomic (see below), and dry cell weight analyses. Dry cell weight (DCW) measurements and anaerobic bioreactor fermentations in YPX + phosphate buffer, pH5.5 were performed as previously described [10]. For cell culture experiments to examine the respiration of various carbon substrates (**Fig 6C and 6D**), Y263 cells were grown to log phase in YPD media aerobically, and then shifted into flasks containing 50 mL fresh YPD, YPX or YP-Ethanol media and incubated at 30°C with shaking. After 6 h (for YPD cultures) or 10 h (for YPX or YP-Ethanol) of growth, 10 mL of culture was transferred to sterile test tubes and treated with 10 μL DMSO or 10 μL of 0.5 mg/mL Antimycin A (0.5 μg/mL final concentration, Sigma-Aldrich). Cell density (OD_600_) measurements were made with a Beckman DU720 spectrophotometer. Glucose, xylose, galactose, glycerol, acetate and ethanol concentrations for all experiments were determined by YSI 2700 Select instrument of by high performance liquid chromatography (HPLC) and refractive index detection (RID) [87].

### Specific consumption and ethanol production rate calculations

Cell growth, specific xylose consumption and ethanol productivity rates were calculated with a rate estimation tool (**S1**and **S2 Appendixes**) using cell density (OD_600_ or DCW), extracellular sugar and ethanol concentrations measured by HPLC-RID. The growth and substrate uptake or product secretion rates were determined by fitting the data to different linear equations using linear regression. The linear equations used to estimate growth and uptake rates, depended on whether data was from exponential growth, linear growth, or stationary (*i*.*e*., non-growth) phases. In the exponential (or linear) phase, the cell concentration increases exponentially (or linearly) with time, while in stationary phase the cell concentration remains constant. Mathematical details and instructions on using this rate-estimating tool can be found in **S2 Appendix**.

### Intracellular protein quantification

After 14 or 20 h of culturing in YPX medium aerobically or anaerobically, respectively (see above), 25 mL of cell culture from each flask was transferred to 50 ml conical tubes, centrifuged at 10,000 RCF for 5 minutes at 4°C. Supernatants were decanted, cells were washed and centrifuged in TE buffer (10 mM Tris pH 7.0, 1 mM EDTA, Life Technologies) and cell pellets flash frozen in dry ice-ethanol for storage. Yeast cell pellets were suspended in 6M guanidine hydrochloride (Sigma, St. Louis, MO) with 50 mM Tris pH 8.0 (Sigma, St. Louis, MO), boiled for 5 min, and methanol was added to a final concentration of 90% to lyse cells and precipitate protein. The precipitate was centrifuged at 10,000 RCF for 5 min, decanted, and air-dried. The protein pellet was resuspended in 8 M urea (Sigma, St. Louis, MO) with 100 mM Tris pH 8.0, 10 mM Tris (2-carboxyethyl) phosphine (Sigma, St. Louis, MO), and 40 mM chloroacetamide (Sigma, St. Louis, MO). The sample was diluted to 1.5 M urea with 50 mM Tris pH 8.0, and trypsin was added to a final ratio of 1:20 (enzyme to protein) followed by overnight incubation at ambient temperature. Tryptic peptides were desalted over Strata-X cartridges (Phenomenex, Torrance, CA). Desalted peptides were dried in a speed vac and resuspended in 0.2% formic acid. Peptides were quantified with the Pierce quantitative colorimetric peptide assay kit (Thermo Fisher Scientific, Rockford, IL). For each analysis, 2 μg of peptides were separated across a 30 cm, 75 μm internal diameter (i.d.) column packed with 1.7 μm BEH C18 particles (Waters, Milford, MA) housed in a capillary column heater set to 65°C. Mobile phase A was 0.2% formic acid and B was 0.2% formic acid, 70% ACN. Peptides were eluted with gradient of 5–50% B over 70 or 100 minutes for anaerobic and aerobic samples, respectively, followed by a 100% B wash and re-equilibration with 0% B [88]. Eluted peptides were analyzed on a Thermo Orbitrap Fusion Lumos (Thermo Fisher Scientific, San Jose, CA). Orbitrap survey scans were performed at 60,000 resolving power with an AGC of 10^6^. The most intense precursors were isolated by the quadrupole with width 1 Da and AGC set to 10^4^, and fragmented by higher energy collisional dissociation in the ion-routing multipole with normalized collision energy set to 30. Fragments were analyzed by turbo scan resolution ion-trap ms/ms. Only precursors with z = 2–8 were sampled, cycle time was set to < 2 s, and dynamic exclusion was 5 s. The maximum injection time for each ms/ms was 15 or 25 ms for anaerobic and aerobic samples, respectively. All analysis of the raw data was performed in the MaxQuant software suite version 1.5.2.8 [89, 90]. Default settings were used except, LFQ and matching between runs were enabled, ITMS match tolerance was set to 0.4 Da, and the min ratio count for quantitation was set to 1. Spectra were searched against a *Saccharomyces cerevisiae* Y22-3 protein database [91] and common contaminant database concatenated with the reverse sequences and filtered to 1% FDR at the peptide and protein level using the target-decoy approach using a reverse decoy database [92]. Raw data files for mass spectrometry proteomic data are available at https://chorusproject.org/pages/dashboard.html#/projects/all/1074/experiments (Project ID 1074). Significant differences in protein abundance were identified using edgeR on the protein-level counts, through pairwise strain comparisons [93], taking an FDR < 0.05 as significant. Functional enrichment was assessed using the FunSpec database [94]. Log_2_ fold-change calculations for protein abundances comparing mutant strains and the Y22-3^MR^ parent strain grown in YPX under aerobic and anaerobic conditions are provided in **S6 Appendix**.

### Intracellular metabolite quantification

For analysis of intracellular metabolites from yeast strains cultured in YPX media aerobically or anaerobically, cell samples were captured and harvested as described in [10] with minor changes. 20 mL of cell culture was applied to a filtration manifold unit (Hoefer FH 225V) outfitted with sterile 0.2 μm pore size nylon filters (Whatman), and the cells captured on the filters under vacuum. The filters were then immediately removed, placed in 15 mL conical tubes containing 4 mL ice-cold extraction buffer (acetonitrile-methanol-water, 40:40:20, 0.1% formic acid) and flash frozen. The concentrations of intracellular ribose-5-phosphate, ribulose-5-phosphate, dihydroxyacetone phosphate, glutathione, xylulose-5-phosphate, trehalose, xylose, xylulose, and xylitol were determined as previously described [10]. Quantifications of all other metabolites were performed as described elsewhere [87]. Log_2_ fold-change calculations for metabolite abundances comparing mutant strains and the Y22-3^MR^ parent strain grown in YPX under aerobic and anaerobic conditions are provided in **S6 Appendix**.

## Supporting Information

S1 Fig Epistatic interactions between *hog1Δ* and *isu1Δ* mutations confer rapid aerobic xylose metabolism. The Y22-3^MR^ parent strain was engineered with various combinations of *isu1Δ*, *hog1Δ* and *gsh1Δ* (**A, B**) or *isu2Δ* and *hog1Δ* (**C, D**) mutations and cultured in YPX media aerobically. Extracellular xylose concentrations (**A** and **C**) and cell densities (**B** and **D**) from the cultures at the indicated times are plotted. Values displayed are averages and standard deviations from three independent biological experiments. The asterisks (*) denote statistical significance between the indicated strains and *isu1Δ* single mutant by paired Student’s t-test, *P* < 0.05.
(TIF)

S2 Fig Deletion of *IRA2* and *GRE3* enables aerobic to anaerobic xylose metabolism. Combinations of *gre3Δ*, *ira2Δ* and *sap190Δ* (**A-B**) or *gre3Δ* and *ira1Δ* mutations (**C-D**) were engineered in the Y127^MR^ and Y128^MR^ strains, which also contained aerobically evolved *hog1*, *isu1* and *gsh1* mutations. Engineered strains were then cultured in YPX media anaerobically, and extracellular xylose concentrations (**A, C**) and cell densities (**B, D**) were measured at the indicated times. Values plotted are averages and standard deviations of 2–3 independent biological replicates.
(TIF)

S3 Fig Deletions of *ISU1*, *HOG1*, *GRE3* and *IRA2* are sufficient to increase xylose fermentation rates. Specific ethanol productivity rates in g of ethanol produced•g of dry cell mass^-1^•h^-1^ (**A**) or g of ethanol produced•OD_600_^-1^•h^-1^ (**B**) from the indicated strains cultured in anaerobic YPX media were calculated from three independent biological replicates. Asterisks denote statistically significant differences (*; *P* < 0.05, **; *P* < 0.063) between indicated strains by paired Student’s t-test.
(TIF)

S4 Fig Deletions of *ISU1*, *HOG1*, *GRE3* and *IRA2* are sufficient to increase cell growth and xylose consumption rates in anaerobic bioreactors. Indicated strains were cultured in YPX media in bioreactors continually sparged with 100% N_2_. Specific growth and xylose consumption rates in OD_600_•hr^-1^ (**A**) and g of xylose consumed•OD_600_^-1^•h^-1^ (**B**) from the indicated strains cultured in YPX media. Graphed average values and standard deviations were calculated from two independent biological replicates.
(TIF)

S5 Fig Deletions of *ISU1*, *HOG1*, *GRE3* and *IRA2* are sufficient for anaerobic xylose metabolism in other strain backgrounds. Indicated strains were cultured in YPX media under anaerobic conditions. Average cell growth (**A**), specific xylose consumption (**B**) and ethanol productivity (**C**) rates in cell mass (in OD_600_)•h^-1^, g xylose, consumed or ethanol produced•L^-1^•h^-1^•cell mass (in OD_600_)^-1^, respectively, were calculated from three independent replicates and plotted. Asterisks denote statistically significant differences (*; *p* < 0.05, **; *p* < 0.08) between indicated strains by paired Student’s t-test.
(TIF)

S6 Fig Deletions of *HOG1* and *ISU1* have different effects on the metabolism of other carbon substrates. The schematic diagram in (**A**) displays the routes of catabolism for the indicated carbon substrates through central metabolism. Dashed arrows indicate that multiple biochemical reactions are involved before the substrate enters central metabolism. Bar graphs display cell growth and specific consumption rates for galactose (**B-C**), ethanol (**D-E**) aerobically, and galactose anaerobically (**F-G**) for the indicated strains. Reported values are averages and standard deviations from biological triplicate experiments, and in g substrate consumed or ethanol produced•L^-1^•h^-1^•cell mass (in OD_600_)^-1^. Asterisks denote statistically significant differences (*P* < 0.05) by Student’s t-test.
(TIF)

S7 Fig Global proteomic analysis identified overlapping functional groups with increased or decreased expression in xylose metabolizing strains. Venn diagrams showing overlap in proteins that increased (left) or decreased (right) in expression level for the indicated xylose metabolizing strains relative to control strains under aerobic (**A**) or anaerobic (**B**) conditions with an FDR of 0.05.
(TIF)

S8 Fig Strains with mutations in *IRA2* display altered levels of trehalose biosynthesis enzymes and intracellular trehalose. Schematic diagram trehalose biosynthesis pathways are displayed (**A**). Heat maps display average log_2_ fold differences in trehalose biosynthesis enzymes for the indicated strains relative to Y22-3^MR^ under aerobic (**B**) or anaerobic (**C**) YPX conditions. Bar graphs display average intracellular trehalose concentrations in μm/g of DCW under aerobic (**D**) or anaerobic (**E**) conditions. All average values and standard deviations were calculated from three independent biological replicates.
(TIF)

S9 Fig TCA Cycle metabolite profiles do not correlate with enzyme profiles in xylose consuming strains. Schematic diagram of the TCA Cycle pathway is displayed (**A**). Heat maps display average Log_2_ fold differences in metabolite (**B-C**) and protein (**D-E**) levels for the indicated strains relative to Y22-3^MR^ under aerobic (**B** and **D**) or anaerobic (**C** and **E**) YPX conditions. White boxes indicate strains from which no metabolite was detected. Average Log_2_ fold differences were calculated from three independent biological replicates. 2-OG, 2-oxoglutarate.
(TIF)

S10 Fig The expression profile of glucose-repressed proteins in anaerobic conditions is distinct from that in aerobic conditions. Engineered and evolved strains were cultured in aerobic YPX media and analyzed for intracellular protein and metabolite concentrations. Average Log_2_ intracellular concentrations of mitochondrial translation and respiration proteins (**A**) or hexose transporters and glucose-repressed proteins (**B**) from mutant strains relative to the Y22-3^MR^ parent are shown. White boxes indicate strains for which no corresponding peptides were detected. Relative protein concentrations were calculated from three independent biological replicates are reported.
(TIF)

S1 Table *S*. *cerevisiae* strains and their genotypes used in this study. (DOCX)

S1 Appendix Batch Culture Rate Estimation Tool version 1.0 By Mingyuan Tian, Jennifer Reed Lab, Chemical & Biological Engineering, University of Wisconsin-Madison. (XLSX)

S2 Appendix User Manual for Batch Culture Rate Estimation Tool. (PDF)

S3 Appendix Comparing Protein Overlap—Increasing Protein in YPX aerobic, S7A Fig. (XLSX)

S4 Appendix EdgeR Log2-fold changes and FDRs for pair-wise protein abundance comparisons between yeast strains grown in YPX aerobic. (XLSX)

S5 Appendix EdgeR Log2-fold changes and FDRs for pair-wise protein abundance comparisons between yeast strains grown in YPX anaerobic. (XLSX)

S6 Appendix Log2 normalized values for proteomic and metabolomic data. (XLSX)
