# Correction: Directed Evolution Reveals Unexpected Epistatic Interactions That Alter Metabolic Regulation and Enable Anaerobic Xylose Use by *Saccharomyces cerevisiae*

**DOI:** 10.1371/journal.pgen.1006447

**Published:** 2016-11-09

**Authors:** Trey K. Sato, Mary Tremaine, Lucas S. Parreiras, Alexander S. Hebert, Kevin S. Myers, Alan J. Higbee, Maria Sardi, Sean J. McIlwain, Irene M. Ong, Rebecca J. Breuer, Ragothaman Avanasi Narasimhan, Mick A. McGee, Quinn Dickinson, Alex La Reau, Dan Xie, Mingyuan Tian, Jeff S. Piotrowski, Jennifer L. Reed, Yaoping Zhang, Joshua J. Coon, Chris Todd Hittinger, Audrey P. Gasch, Robert Landick

## Authorship

Dr. Jeff S. Piotrowski should be included in the author byline instead of the Acknowledgments. He should be listed as the seventeenth author, and his affiliation is: DOE Great Lakes Bioenergy Research Center, University of Wisconsin-Madison, Madison, Wisconsin, United States of America. The contribution of this author is as follows: Investigation.

## Competing Interests

The competing interests statement is incorrect. The statement should read: I have read the journal's policy and the authors of this manuscript have the following competing interests: The Wisconsin Alumni Research Foundation has a provisional patent application entitled "Recombinant yeast having enhanced xylose fermentation capabilities and methods of use". TKS and JSP are inventors. MTr, LSP, ASH, KSM, AJH, MS, SJM, IMO, RJB, RAN, MAM, QD, AL, DX, MTi, JLR, YZ, JJC, CTH, APG, and RL have declared that no competing interests exist.

## References

[1] Sato TK, Tremaine M, Parreiras LS, Hebert AS, Myers KS, Higbee AJ, et al (2016) Directed Evolution Reveals Unexpected Epistatic Interactions That Alter Metabolic Regulation and Enable Anaerobic Xylose Use by *Saccharomyces cerevisiae*. PLoS Genet 12(10): e1006372 doi:10.1371/journal.pgen.1006372 27741250 10.1371/journal.pgen.1006372 PMC5065143

