# Supplementary figures and images for: Directed Evolution Reveals Unexpected Epistatic Interactions That Alter Metabolic Regulation and Enable Anaerobic Xylose Use by Saccharomyces cerevisiae
Source: PLoS Genet. 2016 Oct 14;12(10):e1006372. doi: 10.1371/journal.pgen.1006372 (PMC5065143; doi:10.1371/journal.pgen.1006372)

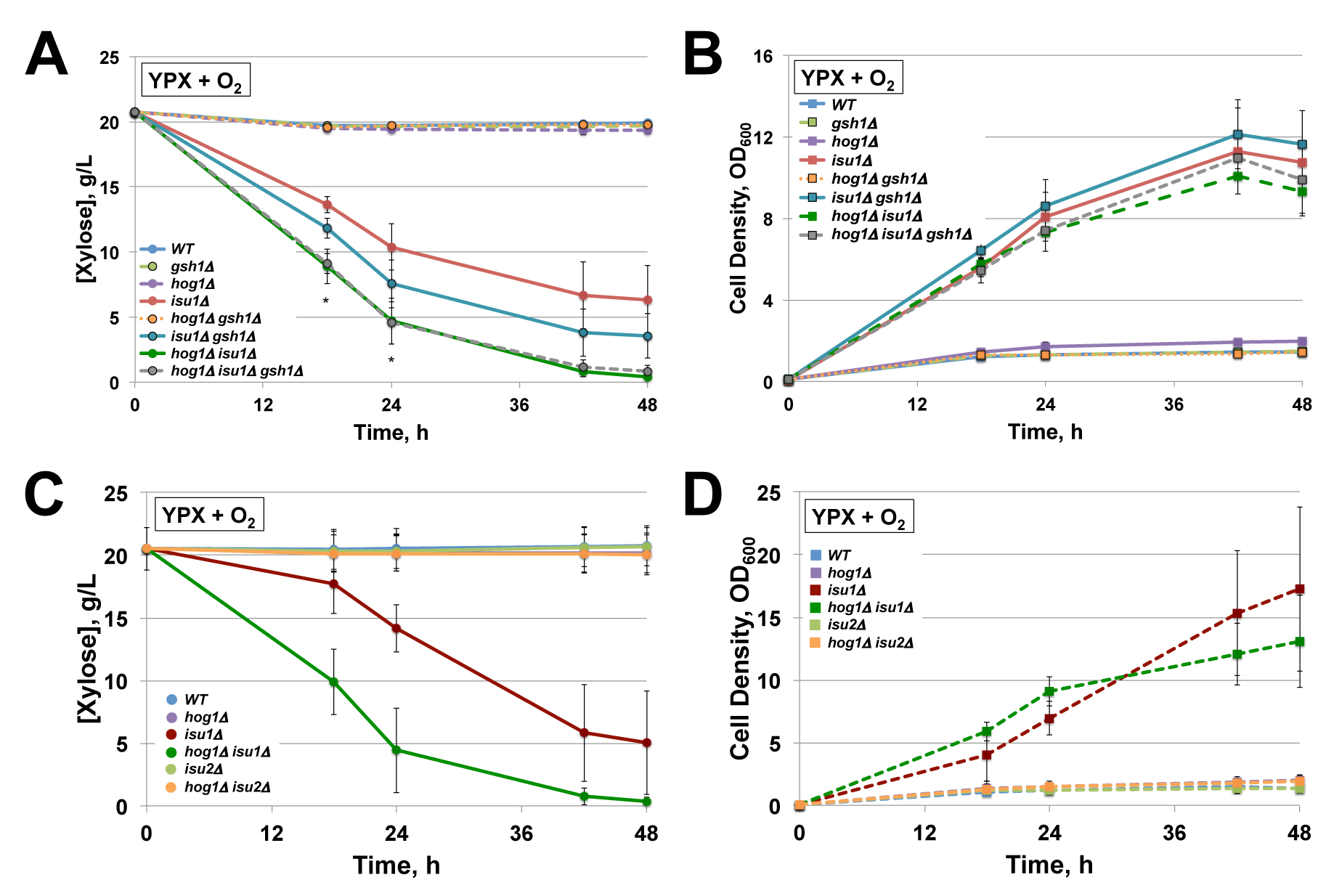

Supplement: S1 Fig — The Y22-3MR parent strain was engineered with various combinations of isu1Δ, hog1Δ and gsh1Δ (A, B) or isu2Δ and hog1Δ (C, D) mutations and cultured in YPX media aerobically. Extracellular xylose concentrations (A and C) and cell densities (B and D) from the cultures at the indicated times are plotted. Values displayed are averages and standard deviations from three independent biological experiments. The asterisks (*) denote statistical significance between the indicated strains and isu1Δ single mutant by paired Student’s t-test, P < 0.05. (TIF) [file pgen.1006372.s001.tif]

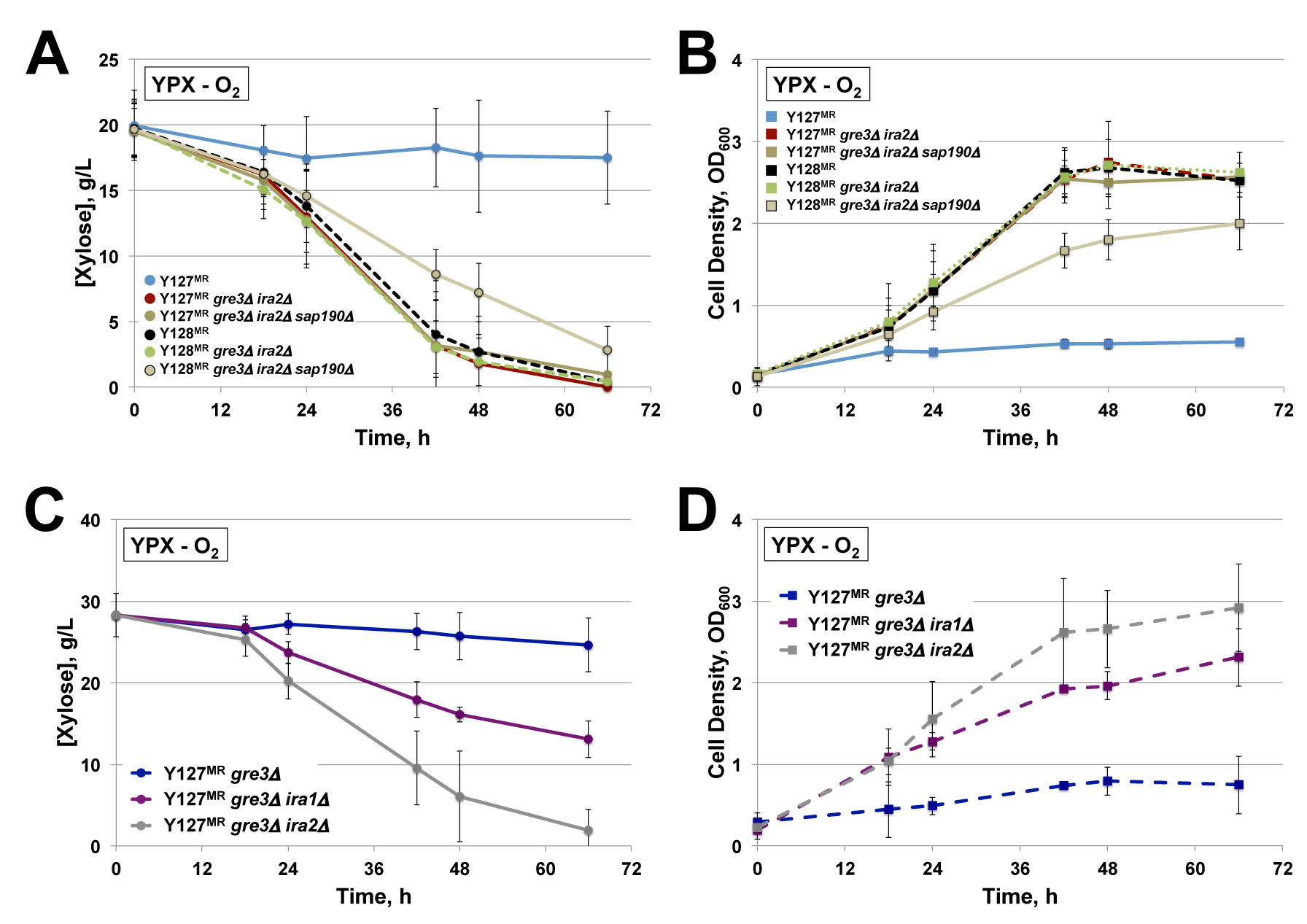

Supplement: S2 Fig — Combinations of gre3Δ, ira2Δ and sap190Δ (A-B) or gre3Δ and ira1Δ mutations (C-D) were engineered in the Y127MR and Y128MR strains, which also contained aerobically evolved hog1, isu1 and gsh1 mutations. Engineered strains were then cultured in YPX media anaerobically, and extracellular xylose concentrations (A, C) and cell densities (B, D) were measured at the indicated times. Values plotted are averages and standard deviations of 2–3 independent biological replicates. (TIF) [file pgen.1006372.s002.tif]

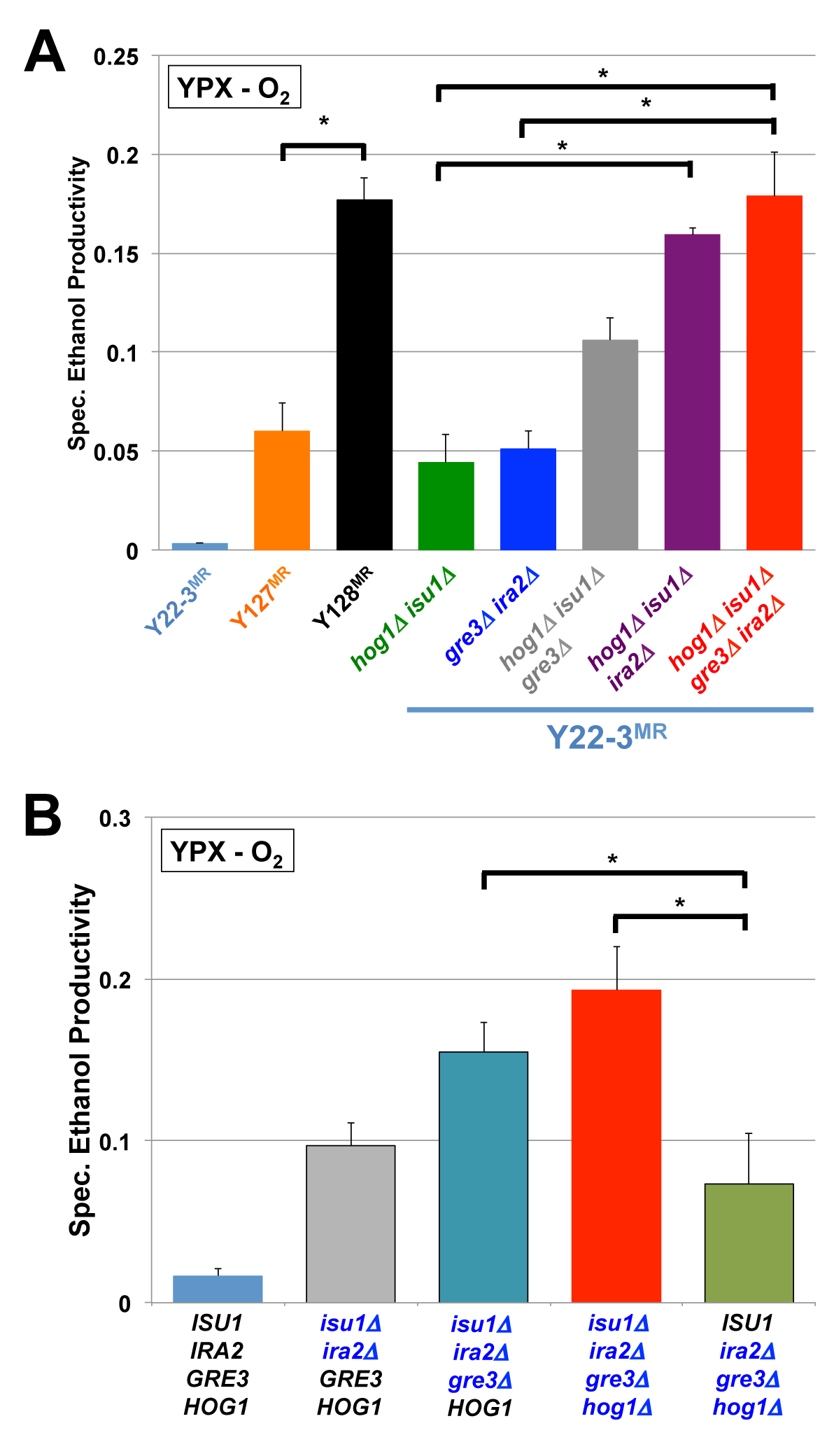

Supplement: S3 Fig — Specific ethanol productivity rates in g of ethanol produced•g of dry cell mass-1•h-1 (A) or g of ethanol produced•OD600-1•h-1 (B) from the indicated strains cultured in anaerobic YPX media were calculated from three independent biological replicates. Asterisks denote statistically significant differences (*; P < 0.05, **; P < 0.063) between indicated strains by paired Student’s t-test. (TIF) [file pgen.1006372.s003.tif]

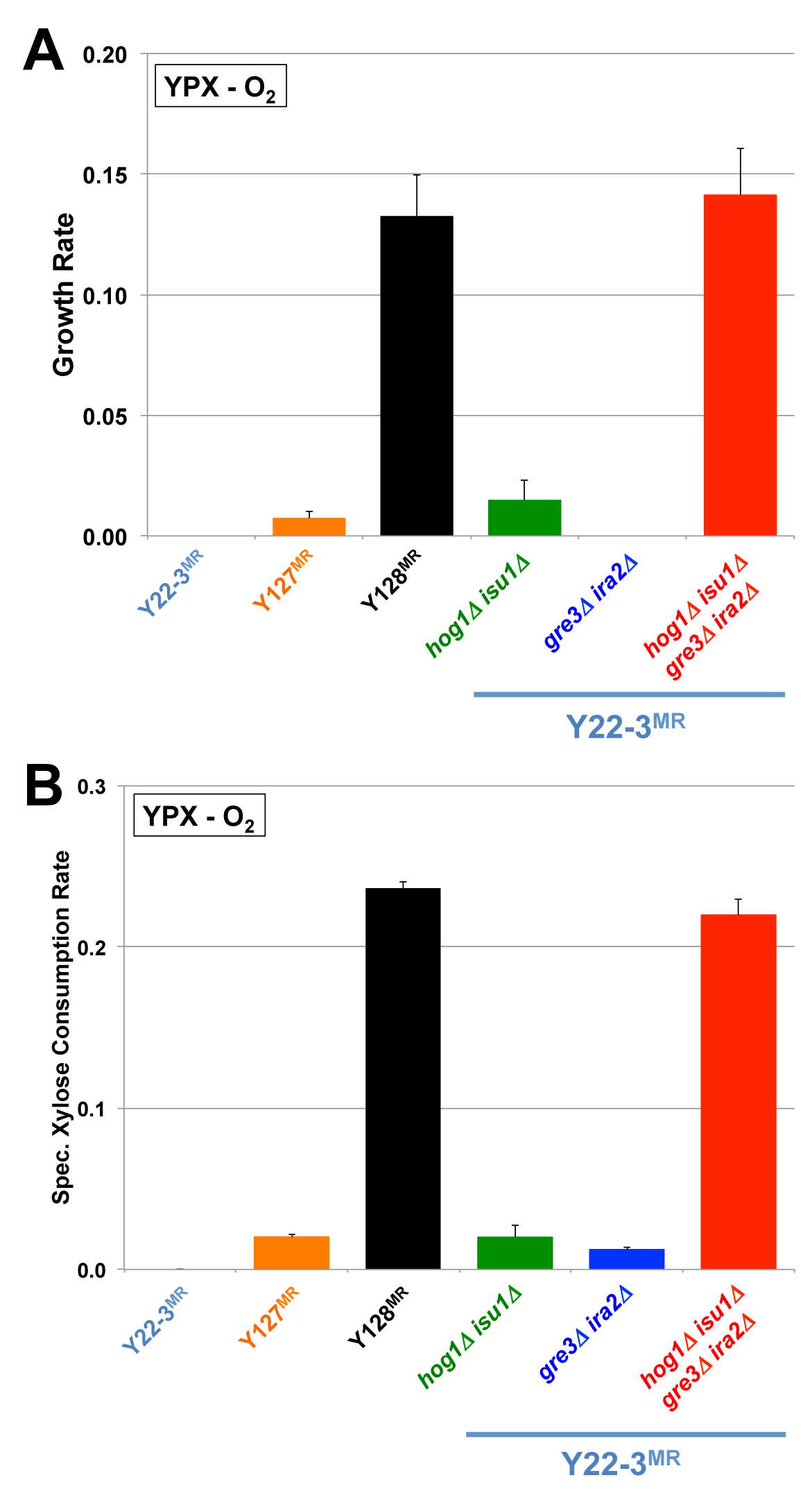

Supplement: S4 Fig — Indicated strains were cultured in YPX media in bioreactors continually sparged with 100% N2. Specific growth and xylose consumption rates in OD600•hr-1 (A) and g of xylose consumed•OD600-1•h-1 (B) from the indicated strains cultured in YPX media. Graphed average values and standard deviations were calculated from two independent biological replicates. (TIF) [file pgen.1006372.s004.tif]

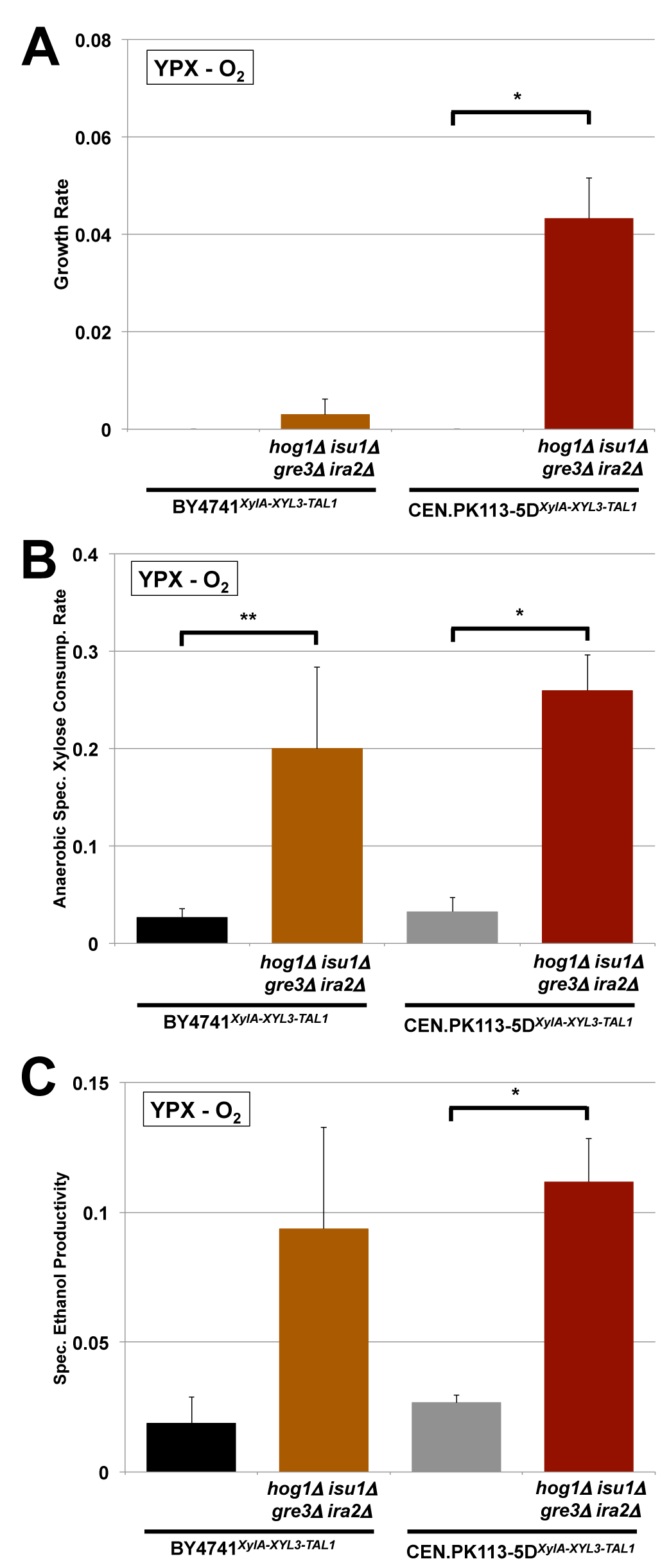

Supplement: S5 Fig — Indicated strains were cultured in YPX media under anaerobic conditions. Average cell growth (A), specific xylose consumption (B) and ethanol productivity (C) rates in cell mass (in OD600)•h-1, g xylose, consumed or ethanol produced•L-1•h-1•cell mass (in OD600)-1, respectively, were calculated from three independent replicates and plotted. Asterisks denote statistically significant differences (*; p < 0.05, **; p < 0.08) between indicated strains by paired Student’s t-test. (TIF) [file pgen.1006372.s005.tif]

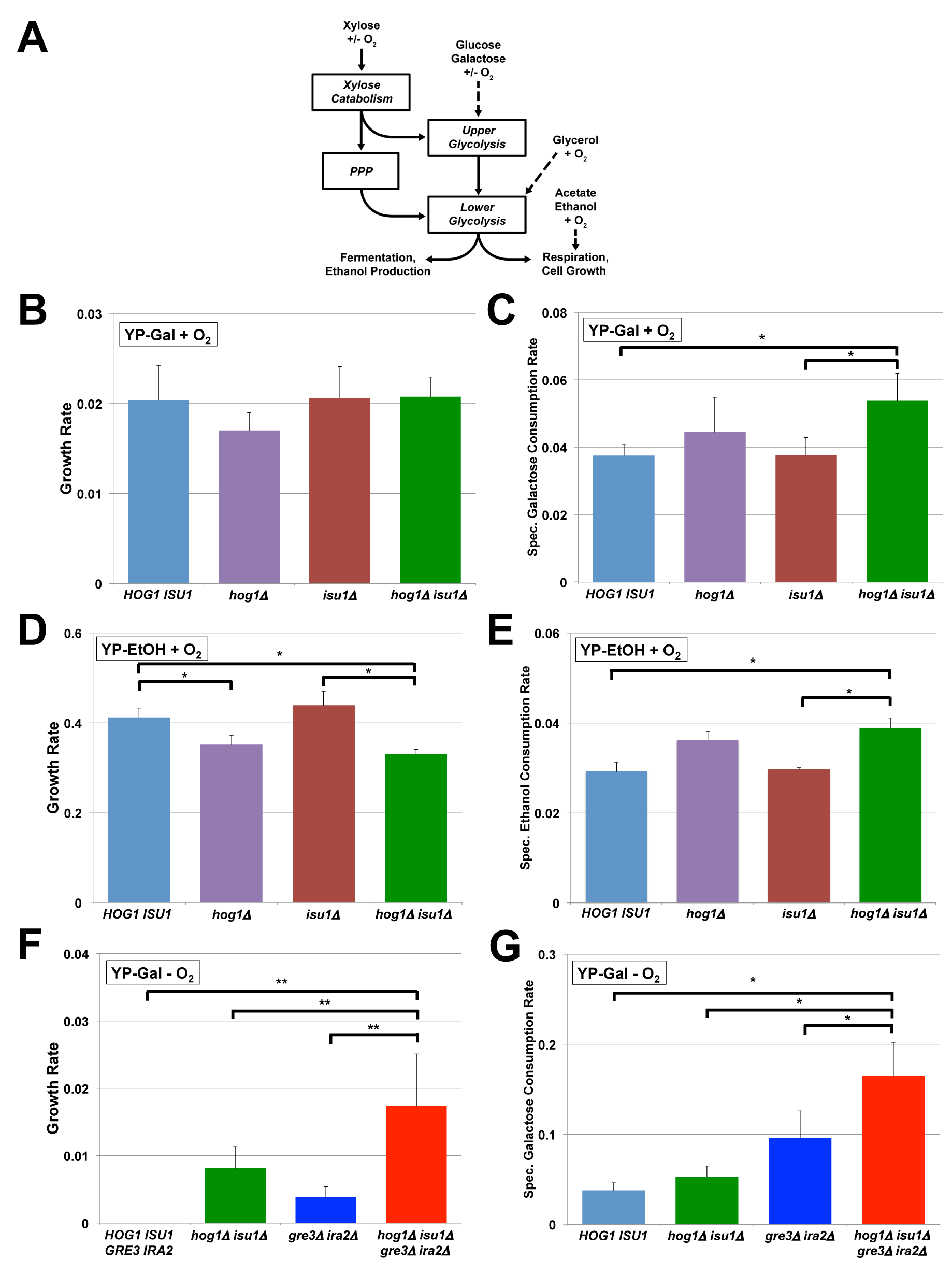

Supplement: S6 Fig — The schematic diagram in (A) displays the routes of catabolism for the indicated carbon substrates through central metabolism. Dashed arrows indicate that multiple biochemical reactions are involved before the substrate enters central metabolism. Bar graphs display cell growth and specific consumption rates for galactose (B-C), ethanol (D-E) aerobically, and galactose anaerobically (F-G) for the indicated strains. Reported values are averages and standard deviations from biological triplicate experiments, and in g substrate consumed or ethanol produced•L-1•h-1•cell mass (in OD600)-1. Asterisks denote statistically significant differences (P < 0.05) by Student’s t-test. (TIF) [file pgen.1006372.s006.tif]

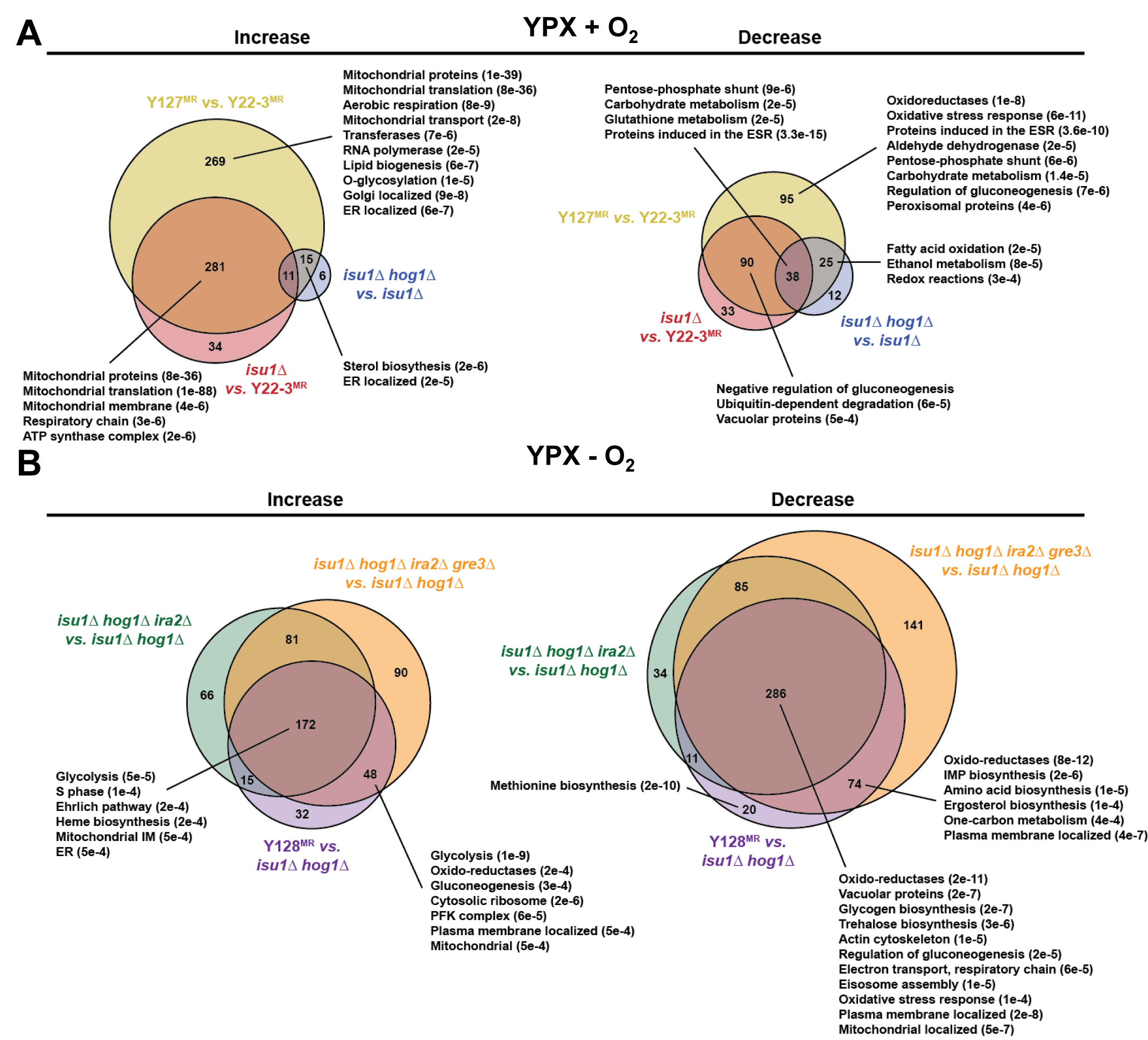

Supplement: S7 Fig — Venn diagrams showing overlap in proteins that increased (left) or decreased (right) in expression level for the indicated xylose metabolizing strains relative to control strains under aerobic (A) or anaerobic (B) conditions with an FDR of 0.05. (TIF) [file pgen.1006372.s007.tif]

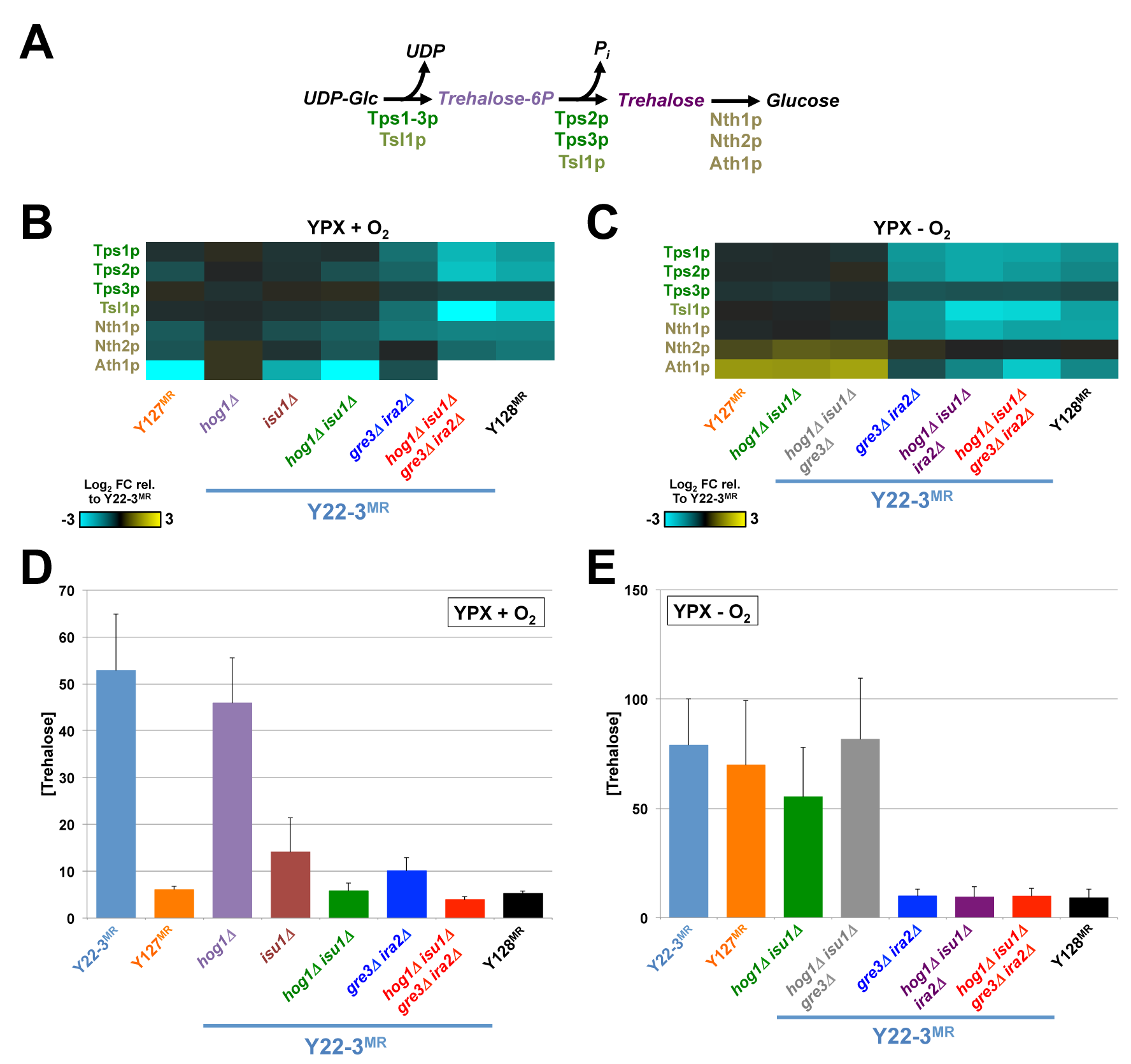

Supplement: S8 Fig — Schematic diagram trehalose biosynthesis pathways are displayed (A). Heat maps display average log2 fold differences in trehalose biosynthesis enzymes for the indicated strains relative to Y22-3MR under aerobic (B) or anaerobic (C) YPX conditions. Bar graphs display average intracellular trehalose concentrations in μm/g of DCW under aerobic (D) or anaerobic (E) conditions. All average values and standard deviations were calculated from three independent biological replicates. (TIF) [file pgen.1006372.s008.tif]

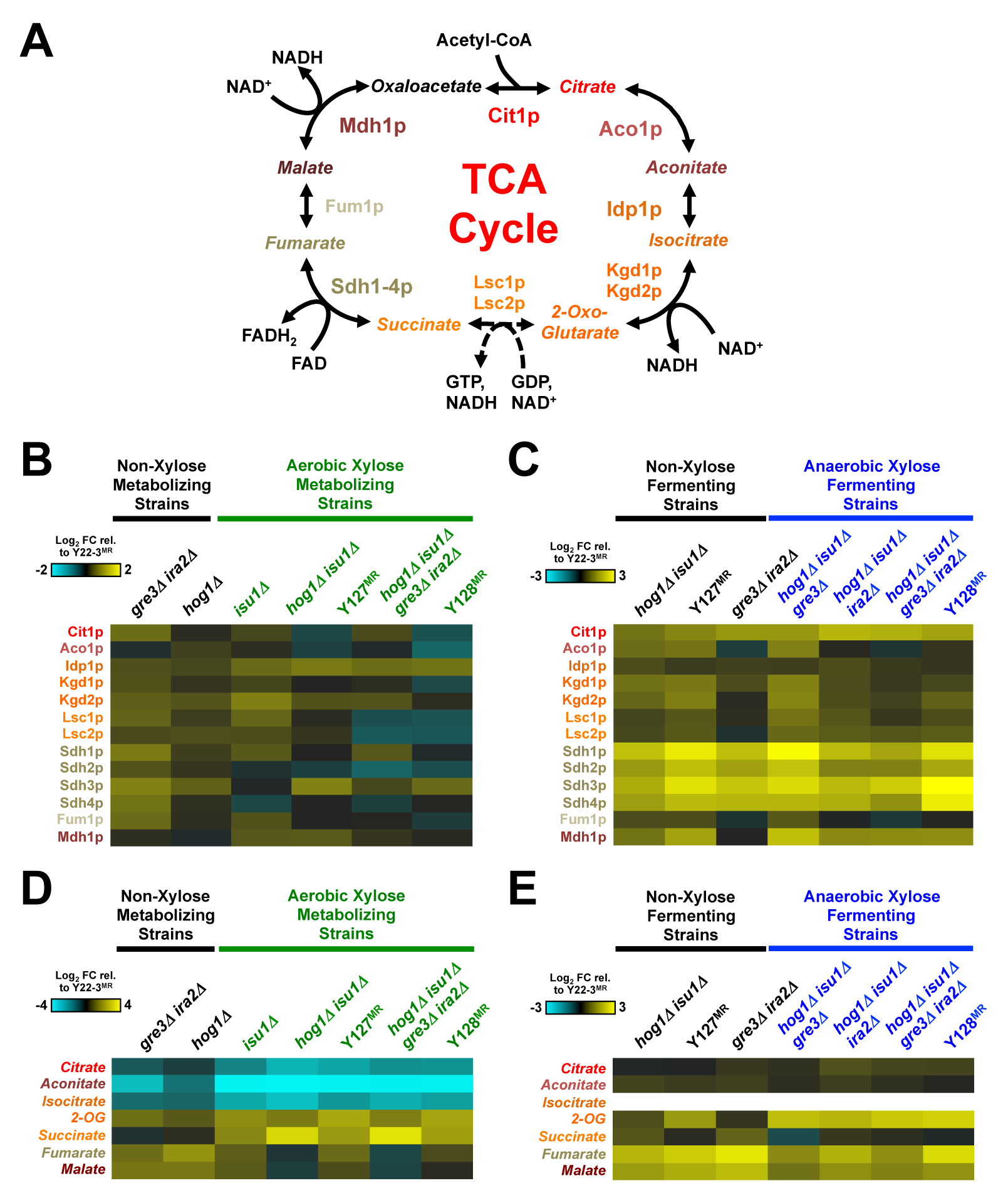

Supplement: S9 Fig — Schematic diagram of the TCA Cycle pathway is displayed (A). Heat maps display average Log2 fold differences in metabolite (B-C) and protein (D-E) levels for the indicated strains relative to Y22-3MR under aerobic (B and D) or anaerobic (C and E) YPX conditions. White boxes indicate strains from which no metabolite was detected. Average Log2 fold differences were calculated from three independent biological replicates. 2-OG, 2-oxoglutarate. (TIF) [file pgen.1006372.s009.tif]

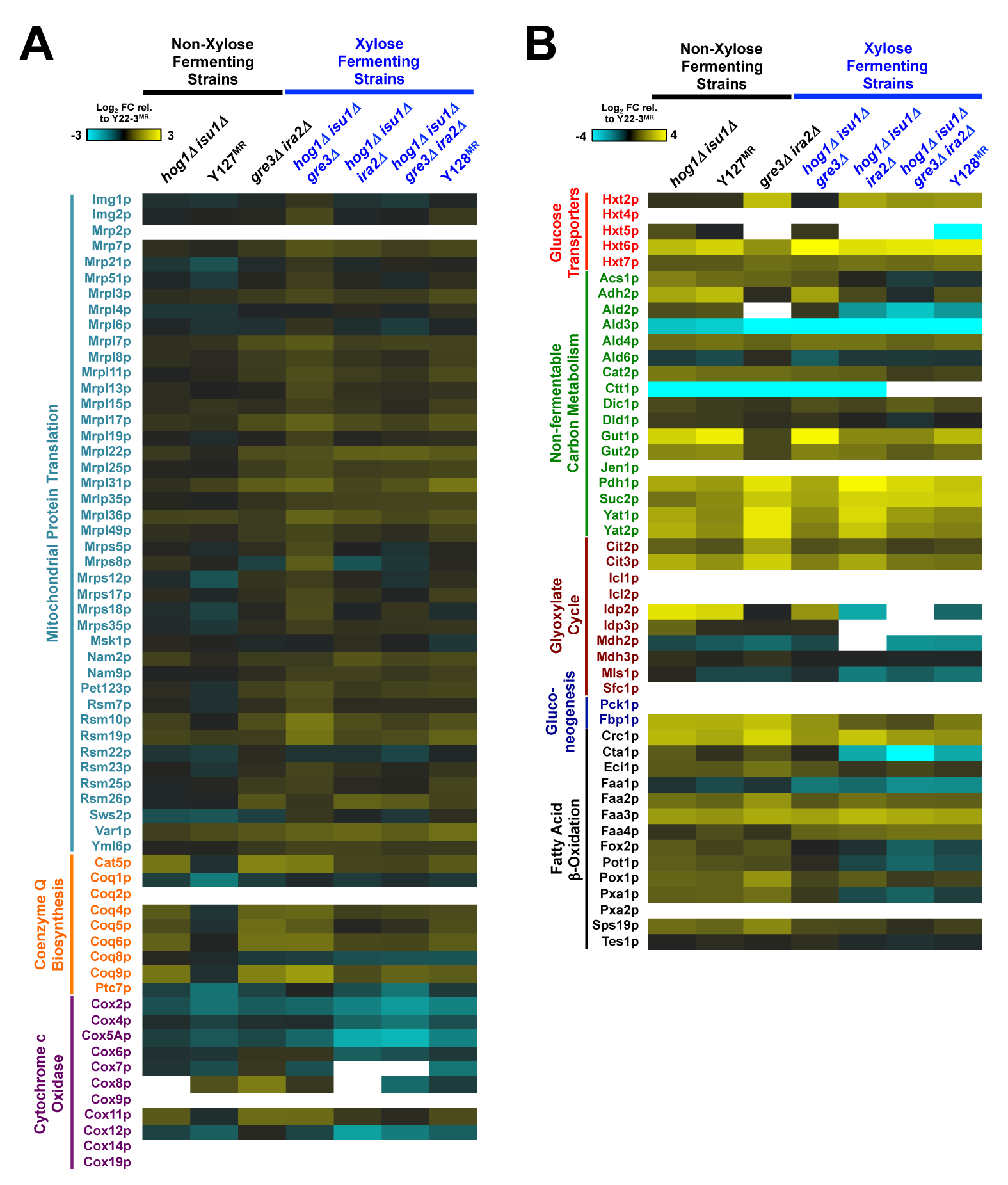

Supplement: S10 Fig — Engineered and evolved strains were cultured in aerobic YPX media and analyzed for intracellular protein and metabolite concentrations. Average Log2 intracellular concentrations of mitochondrial translation and respiration proteins (A) or hexose transporters and glucose-repressed proteins (B) from mutant strains relative to the Y22-3MR parent are shown. White boxes indicate strains for which no corresponding peptides were detected. Relative protein concentrations were calculated from three independent biological replicates are reported. (TIF) [file pgen.1006372.s010.tif]
