# Supplementary material for: Directed Evolution Reveals Unexpected Epistatic Interactions That Alter Metabolic Regulation and Enable Anaerobic Xylose Use by Saccharomyces cerevisiae
Source: PLoS Genet. 2016 Oct 14;12(10):e1006372. doi: 10.1371/journal.pgen.1006372 (PMC5065143; doi:10.1371/journal.pgen.1006372)
Supplement: S1 Table — (DOCX) [file pgen.1006372.s011.docx]

**S1 Table. *S. cerevisiae* strains and their genotypes used in this study.**

| **Genotype** | **Strain names** | **Reference** |
| --- | --- | --- |
| NRRL YB-210 MATa spore with *HOΔ::ScTAL1-CpxylA-SsXYL3-loxP-kanMX-loxP* | GLBRCY22-3 | [10] |
| GLBRCY22-3 MATa, evolved isolate on xylose aerobically | GLBRCY127 | [10] |
| GLBRCY127 MATa, evolved isolate on xylose anaerobically | GLBRCY128 | [10] |
| GLBRCY22-3 with *HOΔ::ScTAL1-CpxylA-SsXYL3-loxP* | GLBRCY22-3^MR^ (GLBRCY36) | [10] |
| GLBRCY127 with *HOΔ::ScTAL1-CpxylA-SsXYL3-loxP* | GLBRCY127^MR^  (GLBRCY132) | [10] |
| GLBRCY128 with *HOΔ::ScTAL1-CpxylA-SsXYL3-loxP* | GLBRCY128^MR^  (GLBRCY133) | [10] |
| GLBRCY127 MATα | GLBRCY156 | This study |
| GLBRCY22-3^MR^ with *hog1Δ::kanMX4* | GLBRCY310 | This study |
| GLBRCY22-3^MR^ with *isu1Δ::loxP* | GLBRCY235 | This study |
| GLBRCY22-3^MR^ with *isu1Δ::loxP,* *hog1Δ::kanMX4* | GLBRCY263 | This study |
| GLBRCY22-3^MR^ with *gre3Δ::LoxP, ira2Δ::LoxP* | GLBRCY212 | This study |
| GLBRCY22-3^MR^ with *isu1Δ::loxP,* *hog1Δ::kanMX4, gre3Δ::LoxP, ira2Δ::LoxP* | GLBRCY286 | This study |
| GLBRCY22-3^MR^ with *gsh1Δ::LoxP-hphMX-LoxP* | GLBRCY227 | This study |
| GLBRCY22-3^MR^ with *hog1Δ::kanMX4, gsh1Δ::LoxP-hphMX-LoxP* | GLBRCY289 | This study |
| GLBRCY22-3^MR^ with *isu1Δ::LoxP, gsh1Δ::LoxP-hphMX-LoxP* | GLBRCY228 | This study |
| GLBRCY22-3^MR^ with *isu1Δ::LoxP, hog1Δ::kanMX4, gsh1Δ::LoxP-hphMX-LoxP,* | GLBRCY292 | This study |
| GLBRCY22-3^MR^ with *isu2Δ::LoxP-bleMX-LoxP* | GLBRCY449 | This study |
| GLBRCY22-3^MR^ with *hog1Δ::kanMX4, isu2Δ::LoxP-bleMX-LoxP* | GLBRCY453 | This study |
| GLBRCY127^MR^ with *gre3Δ::LoxP, ira2Δ::LoxP* | GLBRCY182 | This study |
| GLBRCY127^MR^ with *gre3Δ::LoxP, ira2Δ::LoxP, sap190Δ::LoxP-kanMX-LoxP* | GLBRCY258 | This study |
| GLBRCY128^MR^ with *gre3Δ::LoxP, ira2Δ::LoxP* | GLBRCY163 | This study |
| GLBRCY128^MR^ with *gre3Δ::LoxP, ira2Δ::LoxP, sap190Δ::LoxP-kanMX-LoxP* | GLBRCY259 | This study |
| GLBRCY127^MR^ with *gre3Δ::LoxP-kanMX-LoxP* | GLBRCY146 | [10] |
| GLBRCY127^MR^ with *gre3Δ::LoxP, ira1Δ::LoxP-hphMX-LoxP* | GLBRCY204 | This study |
| GLBRCY36 with *isu1Δ::loxP, hog1Δ::kanMX4, gre3Δ::loxP* | GLBRCY312 | This study |
| GLBRCY36 with *isu1Δ::loxP, hog1Δ::kanMX4, ira2Δ::loxP-HygMX-loxP* | GLBRCY283 | This study |
| GLBRCY22-3^MR^ with *isu1Δ::LoxP, ira2Δ::LoxP-hphMX-LoxP* | GLBRCY242 | This study |
| GLBRCY22-3^MR^ with *hog1Δ::kanMX4, gre3Δ::LoxP, ira2Δ::LoxP* | GLBRCY280 | This study |
| GLBRCY22-3^MR^ with *isu1Δ::LoxP, gre3Δ::LoxP, ira2Δ::LoxP* | GLBRCY243 | This study |
| BY4741 with *HOΔ::ScTAL1-CpxylA-SsXYL3-loxP* | GLBRCY174 | This study |
| CEN.PK113-5D with *HOΔ::ScTAL1-CpxylA-SsXYL3-loxP* | GLBRCY176 | This study |
| GLBRCY174 with *isu1Δ::loxP, hog1Δ::kanMX4, gre3Δ::loxP, ira2Δ::loxP* | GLBRCY276 | This study |
| GLBRCY176 with *isu1Δ::loxP, hog1Δ::kanMX, gre3Δ::loxP, ira2Δ::loxP* | GLBRCY278 | This study |
