# Supplementary material for: Directed Evolution Reveals Unexpected Epistatic Interactions That Alter Metabolic Regulation and Enable Anaerobic Xylose Use by Saccharomyces cerevisiae
Source: PLoS Genet. 2016 Oct 14;12(10):e1006372. doi: 10.1371/journal.pgen.1006372 (PMC5065143; doi:10.1371/journal.pgen.1006372)
Supplement: S2 Appendix — (PDF) [file pgen.1006372.s013.pdf]

User Manual for Batch Culture Rate Estimation Tool

Prepared by Mingyuan Tian and Jennifer L. Reed

This excel worksheet is used to calculate the growth rates and uptake (or secretion) rates in exponential growth, linear growth, and/or stationary (*i.e.*, no growth) phases from concentration data measured in batch culture experiments. The input data are tables of time and OD and time and substrate concentrations.

How to use it?

Be sure to turn on automatic calculation in Excel (this ensures that the cells are updated after you enter your data—you should see the results change after copying in your data):

On a PC with Excel 2010 and 2013: Go to File > Options > Formulas > Workbook Calculation > Automatic.

If the values in the yellow "results" boxes do not update automatically, their values can be updated manually by double-clicking on the corresponding cells and then pressing enter (without changing any of the equations).

The LINEST function from Excel was used to determine the slopes and intercepts (and their standard deviations) for a series of linear least squares regressions (described below).

<u>1. In the "OD vs time" worksheet</u>:

1.1 Paste your time and OD data for the complete experiment into the blue table on the lower left. Make sure to expand the blue table so that it includes all of the data by dragging the lower right corner of the blue table. Evaluate the resulting graphs and decide which time points correspond to exponential growth, linear growth, and stationary (i.e. no growth) phases. Exponential growth should correspond to points that are linear on the ln(OD) versus time graph. Linear growth should correspond to points that are linear on the OD versus time graph. Stationary growth should correspond to points that have an almost constant OD over a period of time.

<u>2. In the "exponential growth phase" worksheet</u>:

2.1 Paste your **time** and **OD** data corresponding to the exponential growth phase under the orange columns labeled 'time(h)' and 'OD'. Since the equations being fit to the data assume the exponential phase starts at time $t = 0$, the times need to be normalized by subtracting the initial time (t_0) from all the times. Similarly, the OD data needs to be transformed by taking the natural log of the OD measurements. To transform the data into $t - t_0$ and $\ln(OD)$ drag the autofill symbol in the blue table immediately to the right of the inputted data. (See **Figure 1**)

27				
28	time(h)	OD	Calculate: t-to	Calculate: lnOD
29	0	0.112	0	-2.189
30	18	0.19		
31	24	0.24		
32				

27				
28	time(h)	OD	Calculate: t-to	Calculate: lnOD
29	0	0.112	0	-2.189
30	18	0.19	18	-1.661
31	24	0.24	24	-1.427
32				

Figure 1: Illustration for dragging the autofill symbol. Before dragging (Left). After dragging (Right).

2.2 Paste your **OD** and **substrate concentration** data corresponding to the exponential growth phase under the orange columns labeled 'OD' and 'Substrate Concentration (g/L)'. Since the equations being fit to the data use the change in OD from the start of the exponential phase, the OD needs to be normalized by subtracting the initial OD ($OD(t_0)$) from all the ODs. To transform the data into $OD(t) - OD(t_0)$ and Substrate Concentration drag the autofill symbol in the blue table immediately to the right of the inputted data.

2.3 After updating the values in these two blue tables, the estimated growth and substrate uptake rates can be found in the yellow cells along with their standard deviations. Their corresponding units are reported assuming time was measured in hours, biomass was measured as OD units, and substrate concentration was measured as g/L. To make sure the estimates seem reasonable the following should be checked:

- The estimated initial OD and substrate concentrations (below the yellow boxes) are similar to the measured initial concentrations in the orange tables.
- Both graphs show good linear relationships with the normalized data (R^2 close to 1).

<u>3. In the "linear growth phase" worksheet</u>:

3.1 Paste your **time** and **OD** data corresponding to linear growth phase under the orange columns labeled 'time(h)' and 'OD'. Since the equations being fit to the data assume the linear phase starts at time $t = 0$, the times need to be normalized by subtracting the initial time (t_0) from all the times. To transform the data into $t - t_0$ and OD drag the autofill symbol in the blue table immediately to the right of the inputted data.

3.2 Paste your **time** and **substrate concentration** data corresponding to exponential growth phase under the orange columns labeled 'time' and 'Substrate Concentration (g/L)'. To fit the data to the linear equation the data needs to be transformed. To transform the data into $\frac{\mu}{2} \cdot (t - t_0)^2 + X_0 \cdot (t - t_0)$ and Substrate Concentration drag the autofill symbol in the blue table immediately to the right of the inputted data. Here, μ is the best estimate for the linear growth rate and X_0 is the best estimate for the initial biomass concentration (i.e., OD), which are both found by fitting the data inputted in step 3.1.

3.3 After updating the values in these two blue tables, the estimated growth and substrate uptake rates can be found in the yellow cells along with their standard deviations. Their corresponding units are reported assuming time was measured in hours, biomass was measured as OD units, and substrate concentration was measured as g/L. **Note**: the growth rate units are different in this worksheet than the other worksheets. To make sure the estimates seem reasonable the following should be checked:

- The estimated initial OD and substrate concentrations (below the yellow boxes) are similar to the measured initial concentrations in the orange tables.
- Both graphs show good linear relationships with the normalized data (R^2 close to 1).

<u>4. In the "stationary phase" worksheet:</u>

4.1 Paste your **time** and **OD** data corresponding to stationary growth phase under the orange columns labeled 'time' and 'OD'. Since the equations being fit to the data assume the linear phase starts at time $t = 0$, the times need to be normalized by subtracting the initial time (t_0) from all the times. To transform the data into $t - t_0$ and OD drag the autofill symbol in the blue table immediately to the right of the inputted data.

4.2 Paste your **time** and **substrate concentration** data corresponding to exponential growth phase under the orange columns labeled 'time' and 'Substrate Concentration (g/L)'. Since the equations being fit to the data assume the linear phase starts at time $t = 0$, the times need to be normalized by subtracting the initial time (t_0) from all the times. To transform the data into $t - t_0$ and Substrate Concentration drag the autofill symbol in the blue table immediately to the right of the inputted data.

4.3 After updating the values in these two blue tables, the estimated substrate uptake rates can be found in the yellow cells along with its standard deviation. Their corresponding units are reported assuming time was measured in hours, biomass was measured as OD units, and substrate concentration was measured as g/L. To make sure the estimates seem reasonable the following should be checked:

- The estimated initial OD and substrate concentrations (below the yellow boxes) are similar to the measured initial concentrations in the orange tables.
- The green graph on the left shows a relatively constant OD over time.
- The grey graph on the right shows a good linear relationship with the normalized data (R^2 close to 1).

Equations used to Derive Linear Relationships Used to Estimate Growth or Uptake Rates:

The equations and derivations below describe how cell concentrations and substrate concentrations change during different phases of growth in a constant volume batch reactor.

X = cell concentration (units of OD)
X_0= initial cell concentration at the beginning of the phase (units of OD)
t = time
t_0 = time the phase starts (normally corresponds to the first time point within a growth phase)
μ = growth rate with units of 1/h or OD/h for exponential and linear growth, respectively.
S = substrate concentration (g/L)
S_0= initial substrate concentration at the beginning of the phase
q= substrate consumption rate (g/L/OD/h)

Exponential growth phase equations:

The derivative for the change in biomass concentration during exponential growth in a batch reactor is given by Eq. 1 [1]:

$$\frac{dX}{dt} = \mu X \qquad \text{(Eq. 1)}$$

By integrating Eq. 1, a linear relationship can be derived (Eq. 2) so that doing a linear least squares regression between lnX and $(t - t_0)$ will yield estimates for μ (the slope) and lnX_0 (the intercept).

$$lnX = lnX_0 + \mu(t - t_0) \qquad \text{(Eq. 2)}$$

The derivative for the change in substrate concentration in a batch reactor is given by Eq. 3 [1]:

$$\frac{dS}{dt} = -qX \qquad \text{(Eq. 3)}$$

By integrating Eq. 3, a linear relationship can be derived (Eq. 4) so that doing a linear least squares regression between S and $(X - X_0)$ will yield estimates for q (where $q = -slope \cdot \mu$) and S_0 (the intercept). The standard deviations for μ and the slope were propagated to find the standard deviation for q.

$$S = S_0 - \frac{q}{\mu}(X - X_0) \qquad \text{(Eq. 4)}$$

Linear growth phase equations:

The derivative for the change in biomass concentration during linear growth in a batch reactor is given by Eq. 5:

$$\frac{dX}{dt} = \mu \qquad \text{(Eq. 5)}$$

By integrating Eq. 5, a linear relationship can be derived (Eq. 6) so that doing a linear least squares regression between X and $(t - t_0)$ will yield estimates for μ (the slope) and X_0 (the intercept).

$$X = X_0 + \mu(t - t_0) \quad \text{(Eq. 6)}$$

The derivative for the change in substrate concentration in a batch reactor is given by Eq. 7:

$$\frac{dS}{dt} = -qX \quad \text{(Eq. 7)}$$

By integrating Eq. 7, a linear relationship can be derived (Eq. 8) so that doing a linear least squares regression between S and $\frac{\mu}{2}(t - t_0)^2 + X_0(t - t_0)$ will yield estimates for q (where $q = -slope$) and S_0 (the intercept).

$$S = S_0 - q[\frac{\mu}{2}(t - t_0)^2 + X_0(t - t_0)] \quad \text{(Eq. 8)}$$

Stationary phase equations:

The derivative for the change in biomass concentration during stationary phase in a batch reactor is given by Eq. 9:

$$\frac{dX}{dt} = 0 \quad \text{(Eq. 9)}$$

By integrating Eq. 9, a linear relationship can be derived showing X is a constant and is independent of time. For this phase, the average X in that phase is represented as $\bar{X}$.

The derivative for the change in substrate concentration in a batch reactor is given by Eq. 10:

$$\frac{dS}{dt} = -qX = -q\bar{X} \quad \text{(Eq. 10)}$$

By integrating Eq. 10, a linear relationship can be derived (Eq. 11) so that doing a linear least squares regression between S and $(t - t_0)$ will yield estimates for q (where $q = -slope \cdot \bar{X}$) and S_0 (the intercept). The standard deviations for $\bar{X}$ and the slope were propagated to find the standard deviation for q.

$$S = S_0 - q\bar{X}(t - t_0) \quad \text{(Eq. 11)}$$

References:

1. Varma, A., and B.O. Palsson. 1994. Stoichiometric flux balance models quantitatively predict growth and metabolic by-product secretion in wild-type *Escherichia coli* W3110. *Appl. Environ. Microbiol.* 60:3724–3731.
